# Analyzing the genes related to nicotine addiction or schizophrenia via a pathway and network based approach

**DOI:** 10.1038/s41598-018-21297-x

**Published:** 2018-02-13

**Authors:** Ying Hu, Zhonghai Fang, Yichen Yang, Dekai Rohlsen-Neal, Feng Cheng, Ju Wang

**Affiliations:** 1 School of Biomedical Engineering, Tianjin Medical University, Tianjin, 300070 China; 2 Department of Pharmaceutical Science, College of Pharmacy, University of South Florida, Tampa, FL 33612 USA

## Abstract

The prevalence of tobacco use in people with schizophrenia is much higher than in general population, which indicates a close relationship between nicotine addiction and schizophrenia. However, the molecular mechanism underlying the high comorbidity of tobacco smoking and schizophrenia remains largely unclear. In this study, we conducted a pathway and network analysis on the genes potentially associated with nicotine addiction or schizophrenia to reveal the functional feature of these genes and their interactions. Of the 276 genes associated with nicotine addiction and 331 genes associated with schizophrenia, 52 genes were shared. From these genes, 12 significantly enriched pathways associated with both diseases were identified. These pathways included those related to synapse function and signaling transduction, and drug addiction. Further, we constructed a nicotine addiction-specific and schizophrenia-specific sub-network, identifying 11 novel candidate genes potentially associated with the two diseases. Finally, we built a schematic molecular network for nicotine addiction and schizophrenia based on the results of pathway and network analysis, providing a systematic view to understand the relationship between these two disorders. Our results illustrated that the biological processes underlying the comorbidity of nicotine addiction and schizophrenia was complex, and was likely induced by the dysfunction of multiple molecules and pathways.

## Introduction

Schizophrenia is a severe psychiatric disorder with 1% of the life-time prevalence in the general population^1,2^. Patients suffering from schizophrenia may show protean manifestations including auditory hallucinations, weird delusions, significant social withdrawal, difficulty in learning/memory retention, and disorganized speech^3^. Currently, there is still no effective treatment for schizophrenia, and those available largely consist in the using of antipsychotic drugs combined with psychological therapies and other approaches. Thus, developing better approaches for schizophrenia treatment remains a pressing task for public health^1^.

Epidemiological studies have shown that individuals with schizophrenia have a higher incidence of substance use as compared to the general population^4–7^. Especially, there is a high prevalence of tobacco smoking among those suffering from the disorder. It has been found that more than 80% of individuals with schizophrenia smoke and are nicotine dependent, compared to the smoking rate of about 25% in the normal individuals^8^. Also, patients suffering from the disorder often smoke high-tar cigarettes and extract more nicotine per cigarette than the common smokers^9^. Moreover, compared to smokers without mental illness, people with schizophrenia face additional challenges, making it more difficult for them to quit smoking^10,11^. Thus, exploring the neurobiological mechanisms that contribute to comorbid nicotine use in schizophrenics is necessary to understand the aetiology and pathogenesis of schizophrenia, and will be helpful for developing more effective therapeutic strategies to prevent and treat the two diseases.

From another prospective, the prevalence of smoking among patients with schizophrenia implicates that some shared neurobiological processes may be responsible for the co-occurrence of the two disorders. One hypothesis is that the use of tobacco in schizophrenic is mainly driven by self-medication since some psychiatric symptoms of schizophrenia can be relieved by smoking^12–14^. There are two main arguments for this hypothesis, one is that nicotine increases the release of neurotransmitters (e.g., dopamine, glutamate and serotonin) and improves the performance in memory and attention^15,16^; the other is that nicotine intake can decrease the side effects of anti-psychotic drugs^17^. However, a recent study raised an objection with self-medication hypothesis as the authors found that cigarette smoking is not associated with cognitive functioning in first-episode psychosis^18^.

Both genetic and environmental factors play roles in the aetiology and development of schizophrenia and nicotine addiction. Over the years, many susceptibility genes associated with schizophrenia or nicotine addiction have been identified^1,19–21^, some of which are common to both disorders. Nicotine, the main psychoactive ingredient in tobacco and a highly addictive substance, evokes its physiological effects by binding with nicotine acetylcholine receptors (nAChRs) and strengthens reward from brain stimulation. nAChRs also play an essential role in cognitive processes such as memory and learning^22^. Actually, several lines of evidence have shown abnormalities of nAChRs in people with schizophrenia^23,24^. For example, the levels of several types of nAChRs are decreased in the brain tissue of postmortem schizophrenia^25^, which may lead to change in the overall neurotransmitter release^26^ and result in some symptoms of schizophrenia^24^. Accumulating evidences from genetic studies demonstrate an association between schizophrenia and polymorphisms in genes encoding α7 subunit (CHRNA7)^27^, α4 and β2 subunits (CHRNA4, CHRNB2)^28^. In addition, COMT gene, a key enzyme in dopamine degradation, may be involved in nicotine addiction^29^ or schizophrenia^30^, or both^31^. Similarly, polymorphisms in the BDNF, DRD1 and DRD3 genes are associated with nicotine addiction, especially in the schizophrenia populations^32^. Moreover, a recent GWAS analysis demonstrates that there is a significant genetic correlation between schizophrenia and smoking behaviors^33^.

For complex disorders like schizophrenia or nicotine addiction, multiple genes may be involved in their aetiology and development. These genes usually function collaboratively to carry out biological functions^34^. Consistently, as more and more genes potentially involved in the pathogenesis of schizophrenia and nicotine addiction were identified, multiple-gene-based bioinformatics approaches, such as pathway and network analysis, have been employed to explore the biological process underlying the schizophrenia^35^ and nicotine dependence^36^. Specifically, pathway-enrichment analysis tools that essentially evolved from methods for gene expression data analysis can also be used to analyze the genes identified from genetic studies. For example, via pathway-enrichment analysis, Walsh *et al*. found neurodevelopment-related pathways, including neuregulin signaling and glutamate receptor signaling, were overrepresented in genes with structural variants specific to patients with schizophrenia^37^. Similar approaches have also been adopted to uncover the pathways associated with genetic mutations in other disorders, including nicotine addiction and Crohn disease^38–40^. Under such a situation, a systematic comparison aiming at revealing the biochemical processes underlying the genes associated with schizophrenia and nicotine addiction will not only help us to understand the relations of these genes but also provide further insights into the molecular mechanism related to the high prevalence of tobacco use in people with schizophrenia.

In this study, we performed a systematic analysis on genes associated with schizophrenia, nicotine addiction, or both via pathway enrichment analysis and network analysis. By such analyses, we identified the genes, pathways, and protein-protein interaction pairs involved in both disorders. Additionally, the candidate genes associated with both diseases were prioritized based on network and pathway analysis. Finally, a molecular network related to schizophrenia and nicotine addiction was constructed to provide a systematic view on the mechanism underlying the high prevalence of smoking in schizophrenia patients at the molecular level.

## Results

### NA and SCZ candidate gene sets

All the candidate genes for nicotine addiction and schizophrenia disease were retrieved from public resources including published papers and database with high confidence as described in Method section. 52 common genes (Supplemental Tables S1, S2 and S3) were identified from 276 genes in NAgenes and 331 genes in SCZgenes. Among these shared genes, some genes were associated with neurotransmission systems including dopaminergic neurotransmitter system (e.g., DRD1, DRD2, DRD3, DRD4 and DRD5), serotonergic neurotransmitter system (e.g., HTR2A, HTR6, TPH1, and TH), glutamatergic neurotransmitter system (e.g., GRIK2, GRIN1, GRIN2A, GRIN2B, GNAS, GRM7 and SLC1A2) and nicotinic neurotransmitter system (e.g., CHRNA7, CHRNB2 and CHRM5). Some genes including SLC1A2, SLC6A3, SLC6A4 and SLC18A2 were participated in cellular transport system. Besides, drug metabolism related genes could be found in shared genes such as ADH1B, CYP2D6, GSTM1, GSTT1 and MAOA.

In order to uncover a more specific function pattern of candidate genes, we further performed function enrichment analysis of these genes related to both diseases. According to the enrichment results exported by DAVID, 160 biological processes (BP) GO terms were significantly enriched in NAgenes and 167 BP GO terms were enriched in SCZgenes (Supplemental Tables S4 and S5). Among these terms, 113 terms were shared between two diseases. As expected, the enriched terms for both diseases basically consisted of synaptic transmission, transmission of nerve impulse, cell-cell signaling, neurological system process, dopamine metabolic process. The results were consistent with the fact that the nicotine addiction and schizophrenia disease were both complex neural system diseases, which also implied that the candidate genes collected are relatively reliable for the following bioinformatics analysis.

### Pathway enrichment analysis

For NAgenes, 20 significantly enriched pathways were identified (Table 1). Of these pathways, some were associated with synaptic transmission, including dopaminergic synapse, cholinergic synapse, and glutamatergic synapse. Some pathways were associated with drug addiction, such as cocaine addiction, amphetamine addiction, nicotine addiction, morphine addiction, and alcoholism. In addition, pathways including neuroactive ligand-receptor interaction, calcium signaling pathway, and cAMP signaling pathway were also enriched. Two pathways related to metabolism, i.e., tryptophan metabolism and tyrosine metabolism were also significantly enriched.

**Table 1:** Pathways Enriched in Genes Associated with Nicotine Addiction.

Pathways	P-value	P_*BH*_-value	Genes included
Neuroactive ligand-receptor interaction	4.19 × 10^−27^	4.24 × 10^−25^	ADRA2A, ADRB2, AGTR1, CHRM1, CHRM2, CHRM5, CHRNA1, CHRNA10, CHRNA2, CHRNA3, CHRNA4, CHRNA5, CHRNA6, CHRNA7, CHRNB1, CHRNB2, CHRNB3, CHRNB4, CHRND, CHRNG, CNR1, DRD1, DRD2, DRD3, DRD4, DRD5, GABBR1, GABBR2, GABRA2, GABRA4, GABRE, GALR1, GRIK1, GRIK2, GRIN1, GRIN2A, GRIN2B, GRIN3A, GRM7, HRH4, HTR1F, HTR2A, HTR6, NPY1R, NPY2R, NR3C1, OPRD1, OPRM1, PARD3
Cocaine addiction	2.10 × 10^−16^	1.06 × 10^−14^	BDNF, CREB1, CREB5, DDC, DLG4, DRD1, DRD2, GNAS, GRIN1, GRIN2A, GRIN2B, GRIN3A, MAOA, MAOB, PPP1R1B, SLC18A2, SLC6A3, TH
Dopaminergic synapse	4.12 × 10^−12^	1.39 × 10^−10^	ARRB2, COMT, CREB1, CREB5, DDC, DRD1, DRD2, DRD3, DRD4, DRD5, GNAS, GRIN2A, GRIN2B, ITPR2, KCNJ6, MAOA, MAOB, PPP1R1B, PPP2R2B, SLC18A2, SLC6A3, TH
cAMP signaling pathway	2.30 × 10^−11^	4.64 × 10^−10^	ABCC4, ADRB2, BDNF, CAMK4, CHRM1, CHRM2, CREB1, CREB5, DRD1, DRD2, DRD5, GABBR1, GABBR2, GNAS, GRIN1, GRIN2A, GRIN2B, GRIN3A, HTR1F, HTR6, NPY, NPY1R, PDE4D, PPP1R1B, RAPGEF3, RHOA
Amphetamine addiction	1.97 × 10^−11^	4.97 × 10^−10^	CAMK4, CREB1, CREB5, DDC, DRD1, GNAS, GRIN1, GRIN2A, GRIN2B, GRIN3A, MAOA, MAOB, PPP1R1B, SLC18A2, SLC6A3, TH
Chemical carcinogenesis	5.14 × 10^−11^	8.65 × 10^−10^	ADH1B, CHRNA7, CYP1A1, CYP1B1, CYP2A6, CYP2E1, EPHX1, GSTM1, GSTM3, GSTP1, GSTT1, NAT1, NAT2, PTGS2, SULT1A1, UGT1A7, UGT2B10
Drug metabolism	3.07 × 10^−9^	4.42 × 10^−8^	ADH1B, CYP2A6, CYP2B6, CYP2D6, CYP2E1, FMO1, GSTM1, GSTM3, GSTP1, GSTT1, MAOA, MAOB, UGT1A7, UGT2B10
Nicotine addiction	6.07 × 10^−9^	7.67 × 10^−8^	CHRNA4, CHRNA6, CHRNA7, CHRNB2, GABRA2, GABRA4, GABRE, GRIN1, GRIN2A, GRIN2B, GRIN3A
Metabolism of xenobiotics by cytochrome P450	9.81 × 10^−9^	1.10 × 10^−7^	ADH1B, CYP1A1, CYP1B1, CYP2A6, CYP2B6, CYP2D6, CYP2E1, EPHX1, GSTM1, GSTM3, GSTP1, GSTT1, UGT1A7, UGT2B10
Alcoholism	1.76 × 10^−8^	1.77 × 10^−7^	BDNF, CAMK4, CREB1, CREB5, DDC, DRD1, DRD2, GNAS, GRIN1, GRIN2A, GRIN2B, GRIN3A, MAOA, MAOB, NPY, NTRK2, PPP1R1B, SHC3, SLC18A2, SLC6A3, TH
Cholinergic synapse	4.92 × 10^−8^	4.52 × 10^−7^	CAMK4, CHAT, CHRM1, CHRM2, CHRM5, CHRNA3, CHRNA4, CHRNA6, CHRNA7, CHRNB2, CHRNB4, CREB1, CREB5, ITPR2, KCNJ6, KCNQ3
Serotonergic synapse	5.60 × 10^−8^	4.72 × 10^−7^	CYP2D6, DDC, GNAS, HTR1F, HTR2A, HTR6, ITPR2, KCNJ6, MAOA, MAOB, PTGS2, RAPGEF3, SLC18A2, SLC6A4, TPH1, TPH2
Morphine addiction	1.04 × 10^−6^	8.05 × 10^−6^	ARRB1, ARRB2, DRD1, GABBR1, GABBR2, GABRA2, GABRA4, GABRE, GNAS, KCNJ6, OPRM1, PDE1C, PDE4D
Calcium signaling pathway	1.81 × 10^−6^	1.30 × 10^−5^	ADRB2, AGTR1, CAMK4, CHRM1, CHRM2, CHRM5, CHRNA7, DRD1, DRD5, GNAS, GRIN1, GRIN2A, HTR2A, HTR6, ITPR2, NOS2, NOS3, PDE1C
Tryptophan metabolism	1.04 × 10^−5^	7.03 × 10^−5^	ALDH2, CYP1A1, CYP1B1, DDC, MAOA, MAOB, TPH1, TPH2
Glutamatergic synapse	1.34 × 10^−5^	8.47 × 10^−5^	DLG4, GNAS, GRIK1, GRIK2, GRIN1, GRIN2A, GRIN2B, GRIN3A, GRM7, HOMER1, HOMER2, ITPR2, SLC1A2
Tyrosine metabolism	3.80 × 10^−5^	2.13 × 10^−4^	ADH1B, COMT, DBH, DDC, MAOA, MAOB, TH
Estrogen signaling pathway	8.00 × 10^−5^	4.25 × 10^−4^	CREB1, CREB5, ESR1, GABBR1, GABBR2, GNAS, ITPR2, KCNJ6, NOS3, OPRM1, SHC3
Rap1 signaling pathway	6.87 × 10^−4^	3.31 × 10^−3^	CNR1, DRD2, FGF12, FGF14, GNAS, GRIN1, GRIN2A, GRIN2B, ITGB3, MAGI1, PARD3, RAPGEF3, RHOA, SIPA1L2, TEK
Steroid hormone biosynthesis	9.95 × 10^−4^	4.37 × 10^−3^	COMT, CYP17A1, CYP1A1, CYP1B1, CYP2E1, UGT1A7, UGT2B10

For SCZgenes, 23 significantly enriched pathways were identified (Table 2). Similar to NAgenes, most of these pathways were closely related to neuronal functions. Pathways of dopaminergic synapse, glutamatergic synapse and serotonergic synapse were identified, which were in agreement with the dopamine hypothesis^41^, glutamate hypothesis^42^, and serotonergic hypothesis^43^ of schizophrenia. In addition, signaling transduction-related pathways, such as calcium signaling pathway, GABAergic synapse and gap junction, long-term depression, cAMP-mediated signaling, T cell receptor signaling pathway, circadian entrainment, and MAPK signaling pathway were also identified. Furthermore, 5 pathways including cocaine addiction, alcoholism, amphetamine addiction, nicotine addiction, and morphine addiction were enriched in SCZgenes, implying that the pathways related to drug addiction may play important roles in the origin and development of schizophrenia.

**Table 2:** Pathways Enriched by Genes Associated with Schizophrenia.

Pathways	P-value	P_BH_-value	Genes included
Neuroactive ligand-receptor interaction	3.29 × 10^−20^	3.09 × 10^−18^	ADRA1A, CCKAR, CHRM5, CHRNA7, CHRNB2, CNR1, DRD1, DRD2, DRD3, DRD4, DRD5, GABBR1, GABRA1, GABRA6, GABRB2, GABRG2, GABRP, GRIA1, GRIA3, GRIA4, GRID1, GRIK2, GRIK3, GRIK4, GRIN1, GRIN2A, GRIN2B, GRIN2D, GRM3, GRM4, GRM5, GRM7, GRM8, HRH1, HRH2, HTR1A, HTR2A, HTR2C, HTR4, HTR5A, HTR6, HTR7, MCHR1, TAAR6
Dopaminergic synapse	1.01 × 10^−15^	4.73 × 10^−14^	AKT1, ATF6B, CLOCK, COMT, DRD1, DRD2, DRD3, DRD4, DRD5, GNAL, GNAO1, GNAS, GNB3, GRIA1, GRIA3, GRIA4, GRIN2A, GRIN2B, GSK3B, MAOA, PPP1R1B, PPP2R2B, PPP3CC, SLC18A1, SLC18A2, SLC6A3, TH
Cocaine addiction	3.06 × 10^−14^	9.59 × 10^−13^	ATF6B, BDNF, DRD1, DRD2, GNAS, GRIN1, GRIN2A, GRIN2B, GRIN2D, GRM3, MAOA, PDYN, PPP1R1B, SLC18A1, SLC18A2, SLC6A3, TH
Amphetamine addiction	5.97 × 10^−14^	1.40 × 10^−12^	ATF6B, DRD1, GNAS, GRIA1, GRIA3, GRIA4, GRIN1, GRIN2A, GRIN2B, GRIN2D, MAOA, PDYN, PPP1R1B, PPP3CC, SLC18A1, SLC18A2, SLC6A3, STX1A, TH
Glutamatergic synapse	3.52 × 10^−13^	6.61 × 10^−12^	GNAO1, GNAS, GNB3, GRIA1, GRIA3, GRIA4, GRIK2, GRIK3, GRIK4, GRIN1, GRIN2A, GRIN2B, GRIN2D, GRM3, GRM4, GRM5, GRM7, GRM8, PLA2G4A, PLA2G4C, PPP3CC, SLC1A2, SLC1A6
Serotonergic synapse	2.09 × 10^−12^	3.28 × 10^−11^	CACNA1F, CYP2D6, GABRB2, GNAO1, GNAS, GNB3, HTR1A, HTR2A, HTR2C, HTR3A, HTR4, HTR5A, HTR6, HTR7, MAOA, PLA2G4A, PLA2G4C, PTGS2, SLC18A1, SLC18A2, SLC6A4, TPH1
Calcium signaling pathway	4.33 × 10^−12^	5.81 × 10^−11^	ADRA1A, CACNA1F, CCKAR, CD38, CHRM5, CHRNA7, DRD1, DRD5, EGFR, ERBB3, ERBB4, GNAL, GNAS, GRIN1, GRIN2A, GRIN2D, GRM5, HRH1, HRH2, HTR2A, HTR2C, HTR4, HTR5A, HTR6, HTR7, NOS1, PPP3CC
Nicotine addiction	5.38 × 10^−12^	6.33 × 10^−11^	CHRNA7, CHRNB2, GABRA1, GABRA6, GABRB2, GABRG2, GABRP, GRIA1, GRIA3, GRIA4, GRIN1, GRIN2A, GRIN2B, GRIN2D
cAMP signaling pathway	1.66 × 10^−7^	1.73 × 10^−6^	AKT1, BDNF, CACNA1F, DRD1, DRD2, DRD5, FXYD2, GABBR1, GNAS, GRIA1, GRIA3, GRIA4, GRIN1, GRIN2A, GRIN2B, GRIN2D, HTR1A, HTR4, HTR6, NPY, PDE4B, PPP1R1B
Alcoholism	2.45 × 10^−6^	2.09 × 10^−5^	ATF6B, BDNF, DRD1, DRD2, GNAO1, GNAS, GNB3, GRIN1, GRIN2A, GRIN2B, GRIN2D, MAOA, NPY, PDYN, PPP1R1B, SLC18A1, SLC18A2, SLC6A3, TH
Retrograde endocannabinoid signaling	2.24 × 10^−6^	2.11 × 10^−5^	CACNA1F, CNR1, GABRA1, GABRA6, GABRB2, GABRG2, GABRP, GNAO1, GNB3, GRIA1, GRIA3, GRIA4, GRM5, PTGS2
Circadian entrainment	6.03 × 10^−6^	4.36 × 10^−5^	GNAO1, GNAS, GNB3, GRIA1, GRIA3, GRIA4, GRIN1, GRIN2A, GRIN2B, GRIN2D, NOS1, NOS1AP, PER3
Rap1 signaling pathway	8.33 × 10^−5^	4.61 × 10^−4^	AKT1, CNR1, DRD2, EGF, EGFR, FGF1, FGF14, FGF18, FGFR1, GNAO1, GNAS, GRIN1, GRIN2A, GRIN2B, MAGI1, MAGI2, MAGI3, RAPGEF6
Morphine addiction	1.02 × 10^−4^	5.33 × 10^−4^	DRD1, GABBR1, GABRA1, GABRA6, GABRB2, GABRG2, GABRP, GNAO1, GNAS, GNB3, PDE4B
MAPK signaling pathway	1.09 × 10^−4^	5.38 × 10^−4^	AKT1, BDNF, CACNA1F, EGF, EGFR, FAS, FGF1, FGF14, FGF18, FGFR1, FLNB, IL1A, IL1B, MAPK8IP2, NTF3, PLA2G4A, PLA2G4C, PPP3CC, TNF, TP53
Cytokine-cytokine receptor interaction	1.84 × 10^−4^	8.64 × 10^−4^	CSF2RA, CSF2RB, CXCR1, EGF, EGFR, FAS, IL10, IL10RA, IL12B, IL18, IL18R1, IL18RAP, IL1A, IL1B, IL2, IL3, IL3RA, IL4, LTA, TNF
GABAergic synapse	3.52 × 10^−4^	1.50 × 10^−3^	CACNA1F, GABBR1, GABRA1, GABRA6, GABRB2, GABRG2, GABRP, GAD1, GNAO1, GNB3
PI3K-Akt signaling pathway	1.13 × 10^−3^	4.19 × 10^−3^	AKT1, ATF6B, EGF, EGFR, FGF1, FGF14, FGF18, FGFR1, FN1, GNB3, GSK3B, IL2, IL3, IL3RA, IL4, PPP2R2B, RELN, TNXB, TP53, YWHAE, YWHAH, YWHAZ
Phospholipase D signaling pathway	1.5 × 10^−3^	4.53 × 10^−3^	AKT1, CXCR1, EGF, EGFR, GNAS, GRM3, GRM4, GRM5, GRM7, GRM8, PLA2G4A, PLA2G4C
ErbB signaling pathway	1.37 × 10^−3^	4.61 × 10^−3^	AKT1, EGF, EGFR, ERBB3, ERBB4, GSK3B, NRG1, NRG2, NRG3
T cell receptor signaling pathway	1.33 × 10^−3^	4.61 × 10^−3^	AKT1, CD4, CTLA4, GSK3B, IL10, IL2, IL4, ITK, PPP3CC, TNF
Gap junction	1.49 × 10^−3^	4.66 × 10^−3^	DRD1, DRD2, EGF, EGFR, GNAS, GRM5, HTR2A, HTR2C, TUBA8
Long-term depression	2.34 × 10^−3^	6.46 × 10^−3^	GNAO1, GNAS, GRIA1, GRIA3, NOS1, PLA2G4A, PLA2G4C

Comparing the pathways associated with the two diseases, there were 8 pathways specific to nicotine addiction, 11 pathways specific to schizophrenia, and 12 shared pathways. These shared pathways include pathways related to synapse function and signaling transduction (i.e., neuroactive ligand-receptor interaction, dopaminergic synapse, calcium signaling pathway, cAMP signaling pathway, glutamatergic synapse and serotonergic synapse) and pathways related to drug addiction (i.e., nicotine addiction, cocaine addiction, morphine addiction, amphetamine addiction, and alcoholism). This implies that the drug addiction-associated pathways may play an essential role in both nicotine addiction and schizophrenia. Some pathways were only significantly enriched in genes associated with one of the two diseases, but they also included genes involved in the other disorder. For example, retrograde endocannabinoid signaling, a pathway mainly consisting of neuromodulatory endocannabinoids and their receptors, and playing important role in mediating global modulation of synaptic strength, localized short-term associative plasticity and cerebellar long term depression, was identified as enriched pathway for schizophrenia. A close inspection showed that for 20 genes included in this pathway, 12 genes were related to schizophrenia, while 6 genes were related to nicotine addiction, and the other 2 genes were associated with both diseases. Another example is the estrogen signaling, a pathway mainly consisting of hormone estrogen and its receptors. Besides its function in the reproductive system, this pathway is also involved in multiple biological processes in the peripheral and central nervous systems and plays important roles in cognition, depression, homeostasis, pain processing, and other associated neuronal functions. The estrogen signaling is significantly enriched in genes related to nicotine addiction, but for 15 genes included in this pathway, 9 were specific to nicotine addiction and 4 to schizophrenia, as well as 2 common genes for both diseases.

### Predicting new candidate genes related to schizophrenia and nicotine addiction

**Figure 1:**
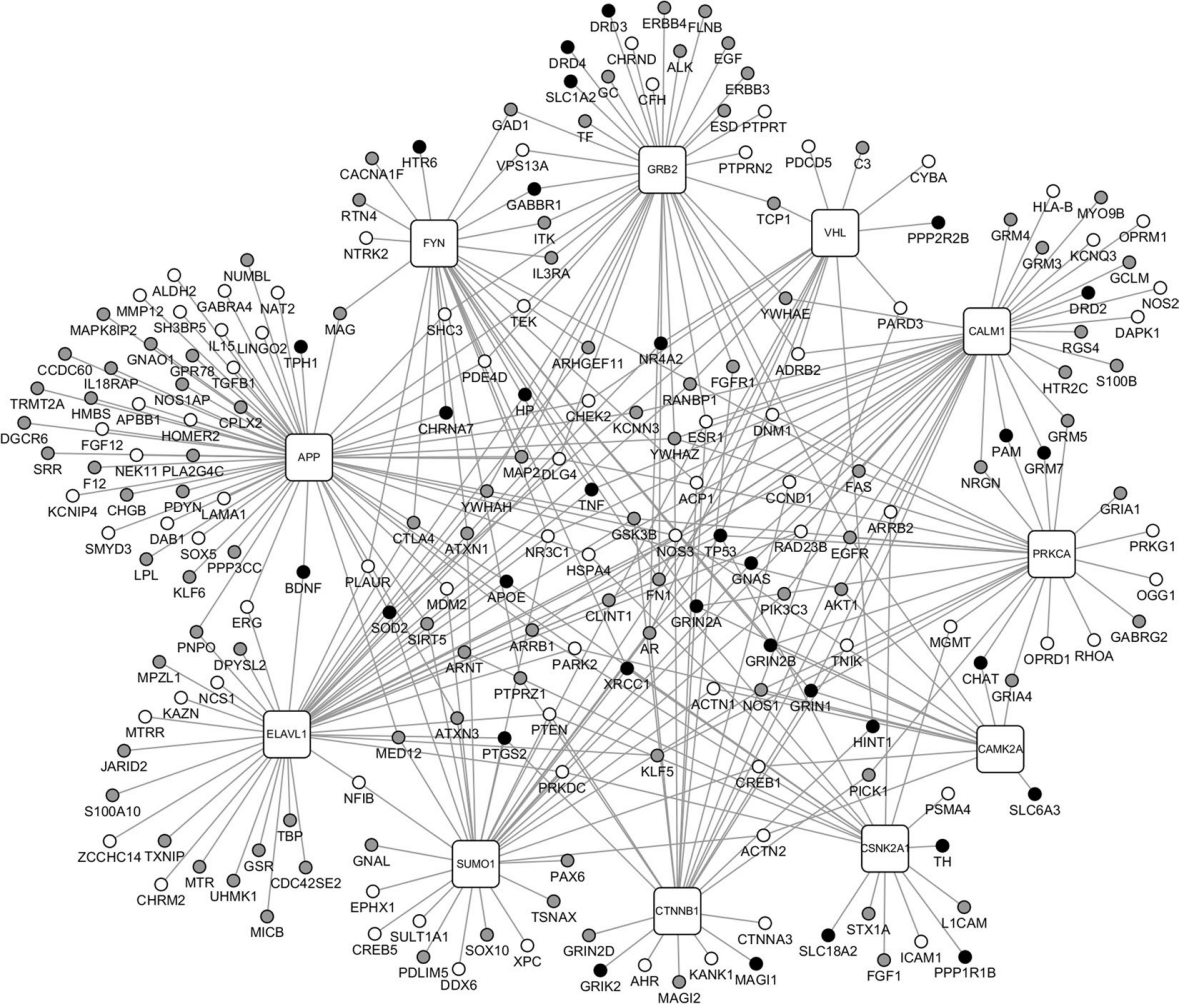
The predicted candidate genes and genes interacted with them in human PPI. Nodes shown in white, grey, and black represent genes associated with schizophrenia, nicotine addiction, or both diseases, respectively. The round rectangle nodes represent genes predicted to be associated with schizophrenia and nicotine addiction based on their network feature.

New genes potentially related to nicotine addiction and schizophrenia could be predicted by analyzing the susceptibility genes associated with the two diseases via the human PPI network. NAgenes were mapped to the PPI network and a NA-specific sub-network with 4,161 nodes was retrieved. The sub-network consists of 247 seed nodes (genes mapped to PPI network) and 3,914 direct neighbor nodes (genes in PPI that were directly connected to one or more seed nodes), and 8,524 interactions. Similarly, the SCZ-specific sub-network consisted of 4,724 nodes (306 seed nodes and 4,418 direct neighbor nodes) and 9,474 interactions. There were 1,388 shared nodes and 1,766 shared interactions between these two sub-networks. Among the shared nodes, 91 genes were from NAgenes, 102 genes were from SCZgenes, and 51 genes were common genes for both diseases. There were 1,145 first neighbor nodes in the shared nodes, from which we identified the new potential risk factors via the ‘guilt-by-association’ principle^44^. That is, a node tends to participate in the same or similar cellular functions if the majority of its neighbors in the interactome network associated with specific cellular functions (e.g., a certain disease or phenotype). In our case, 11 genes directly interacted with 5 or more of the 51 common genes were considered as new risk genes associated with the two diseases, including proto-oncogene tyrosine-protein kinase Fyn (FYN), protein kinase C alpha (PRKCA), ELAV-like protein 1 (ELAVL1), casein kinase II subunit alpha (CSNK2A1), calmodulin 1 (CALM1), von Hippel–Lindau tumor suppressor (VHL), small ubiquitin-related modifier 1 (SUMO1), growth factor receptor-bound protein 2 (GRB2), catenin beta-1 (CTNNB1), calcium/calmodulin-dependent protein kinase type II alpha chain (CAMK2A) and amyloid precursor protein (APP). The network constructed by 11 new candidate genes and their interacted candidate genes was shown in Fig. 1.

## Discussion

For complex diseases, the phenotype or disease status is rarely a straight-forward dysfunction in a specific gene or pathway, but rather is an interplay between multiple genes that collectively induces the dysfunctional effects on multiple biological pathways and causes the clinic outcome eventually^45,46^. Both nicotine addiction and schizophrenia are complex diseases with numerous genes involved in their aetiology and development; meanwhile, the high prevalence of cigarette smoking in schizophrenia patients implicates a close connection between the two disorders. The common susceptibility genes related with two diseases may contribute to the association between the diseases^46^. Therefore, an analysis on the genes related to nicotine addiction and schizophrenia can help to uncover their relationship at the molecular level.

Pathway enrichment analysis revealed that several signaling pathways related to neuronal function were highly enriched in genes related to nicotine addiction or schizophrenia. 12 significantly enriched pathways shared by both diseases can be divided into two groups: the neurodevelopment-related pathways (including neuroactive ligand-receptor interaction, dopaminergic synapse, cAMP signaling pathway, calcium signaling pathway, glutamatergic synapse, serotonergic synapse and rap1 signaling pathway) and the drug addiction-related pathways (including cocaine addiction, nicotine addiction, morphine addiction, amphetamine addiction and alcoholism). For the neurodevelopment-related pathways, pathways like neuroactive ligand-receptor interaction, cAMP signaling pathway and calcium signaling pathway have been implicated in diseases including nicotine addiction and schizophrenia. Three pathways associated to non-cholinergic synapse were included and these results were consistent with prior knowledge about the mechanism of brain diseases^47^. In detail, stimulating the nicotine acetylcholine receptors (nAChRs) induces changes in intracellular Ca^2+^ concentration, which further regulates the release of neurotransmitters such as dopamine, glutamate, and serotonin. These neurotransmitters are essential in the pathogenic development and treatment of both nicotine addiction^48^ and schizophrenia^49^. For example, dopamine, the most important component in the brain reward system, plays a key role in reinforcing effects of nicotine^50^. Meanwhile, even though some symptoms of schizophrenia attribute to a disturbed and hyperactive dopaminergic signal transduction according to the dopamine hypothesis of schizophrenia^41^, smoking stimulates the dopaminergic activity in the brain by increasing the concentration of dopamine and in turn may contribute to antidepressant effects of nicotine molecules in schizophrenia patients. Accordingly, one interpretation about the high nicotine addiction and schizophrenia comorbidity suggests that smoking represents an attempt to self-medicate in schizophrenic patients, aiming to reducing extrapyramidal symptoms associated with antipsychotic treatment, and alleviating cognitive deficits associated with schizophrenia^51^. In addition to dopamine, the glutamate and serotonin also play a role in ‘the self-medication’ of schizophrenia patients who smoke^52^. Coupled with the development of atypical anti-psychotic medications, they have the ability modulate the dopamine neurotransmission release^53,54^ and reinforce the effect of nicotine. Except the three basal pathways and the three pathways related to non-cholinergic synapse, rap1 signaling pathway was significantly enriched in both nicotine addiction and schizophrenia. This pathway was found to be involved in synaptic plasticity, exciting, learning and memory with inhabiting L-type calcium channel-dependent neurotransmitters release^55,56^.

We identified five significantly enriched pathways-related to drug addiction, including cocaine addiction, nicotine addiction, morphine addiction, amphetamine addiction, and alcoholism, were involved in both diseases, which may imply that the drug addiction related pathways play important roles in the development of schizophrenia. On one hand, our result may provide some clues on the high co-occurrence of substance addiction and schizophrenia reported by previous studies^57–59^ from a perspective of pathway dysfunction. On the other hand, our results show that the membrane receptor proteins participating in drug addiction pathways, such as nAChRs and μ opioid receptor (MOR), may be the potential target for therapeutic treatment for schizophrenia. In fact, the participation of several subtypes of neuronal nAChRs has been explored as therapeutic targets for treatment of various diseases such as pain, schizophrenia and Alzheimer’s disease^60^. Earlier study has found that the expression level of MOR mRNA is higher in schizophrenia, which may contribute to suppressed GABA neuron activity in prefrontal cortical which result in cognitive impairments in schizophrenia^61^. Besides, a recent study demonstrated that genetic variation of MOR in schizophrenics was closely related to their smoking differences^62^. However, the utilization of MOR in the therapeutic treatment for both nicotine addiction and schizophrenia still requires further investigation.

Through network analysis, we found that a large number of dysfunctional PPI pairs involved in nicotine addiction or schizophrenia, and several common PPI pairs co-occurrence in both diseases were employed to construct a common sub-network for both diseases. By calculating the degree parameter of each node in this common sub-network, we identified 11 susceptible genes potentially involved in both diseases. Most of these genes have been reported to be associated with nicotine addiction, schizophrenia or both diseases through various experimental approaches (Supplemental Table S6). Taking FYN for example, as a member from the tyrosine kinase in Src family, this gene is an important component for various cellular processes including synaptic plasticity^63^. It is abundantly expressed in the hippocampus, cerebral cortex, and thalamus and has a key role in long-term potentiation (LTP) and in the relation of LTP to spatial learning and memory^64^. This gene has been proved to be associated with both nicotine addiction and schizophrenia^65–67^; e.g., it can increase the activity of NMDA receptor by regulating the glutamatergic signaling and further influences the performance in schizophrenics, and is implicated in the development of nicotine addiction via interaction with α7 nAChR. In addition, for some of these novel genes (e.g., CALM1 and ELAVL1), although no available study has demonstrated their connection with schizophrenia or nicotine addiction directly, they have been identified as risk factors for other mental disorders including autism and drug addictions. Besides, these genes were included in multiple pathways enriched in the genes associated with one or both diseases. For example,CALM1 is a common component for 6 enriched pathways such as alcoholism, amphetamine addiction, calcium signaling pathway, cAMP signaling pathway, dopaminergic synapse and Rap1 signaling pathway. The involvement of these new susceptible genes pathways associated with both nicotine addiction and schizophrenia provides further evidence for their connection with the two diseases. Thus, these genes may be new candidates for further exploration.

**Figure 2:**
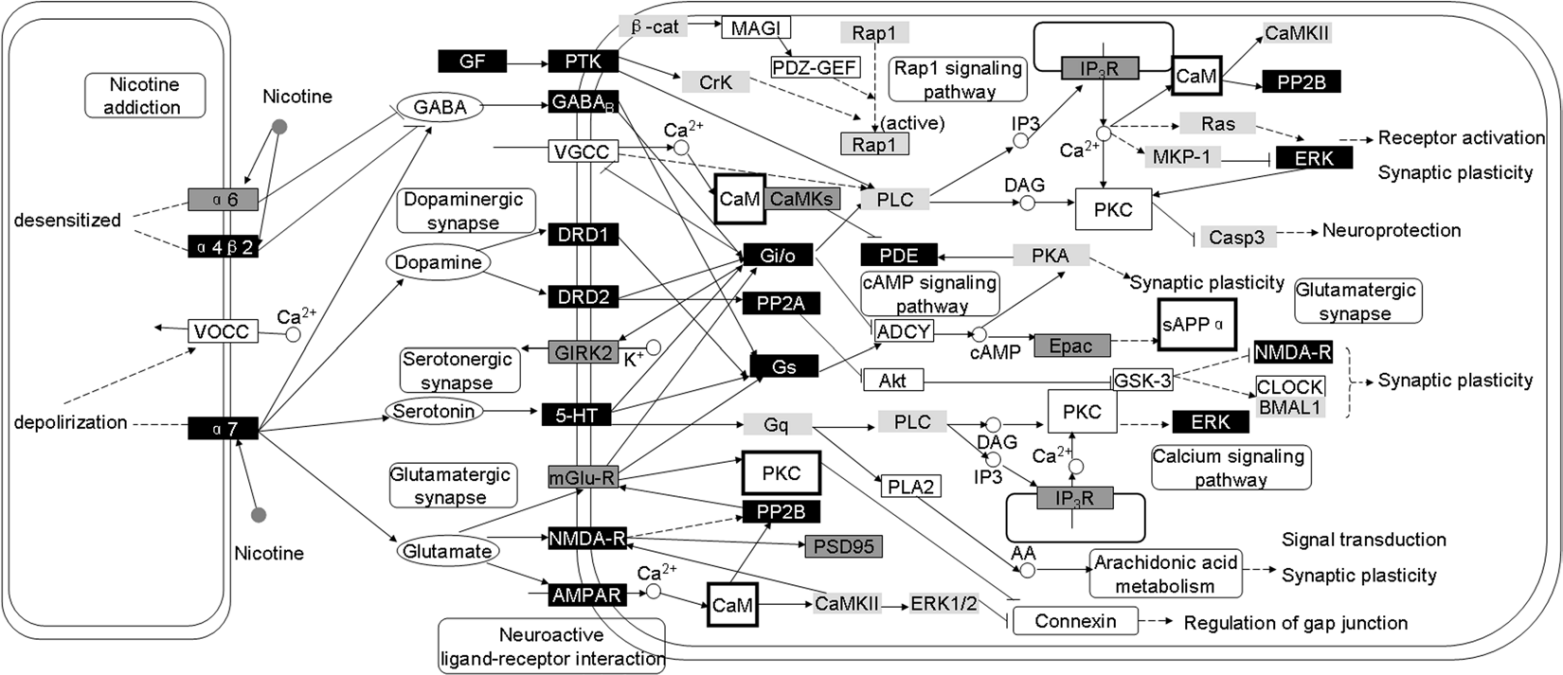
A systematic network of the molecular mechanism underlying schizophrenia and nicotine addiction. This network was built based on the results of pathway analysis and network analysis. Nodes shown in white, grey, and black rectangles represent genes related to schizophrenia, nicotine addiction, or both diseases, respectively. The predicted candidate genes are shown by larger rectangles with bold border. Ellipses represent neurotransmitters like GABA, serotonin, dopamine and glutamate. Dashed and solid lines represent indirect and direct regulations; lines with arrow and spot represent activation and inhibition, respectively.

Based on the results from pathway analysis and network analysis, the relationship of nicotine addiction and schizophrenia could be summarized into a schematic network (Fig. 2). This network consists of a number of key genes, transmembrane receptors and neurotransmitters associated with the two diseases. In addition, the common pathways enriched for two diseases including neurotransmitters receptor signaling transduction pathways and intracellular signaling transduction cascades were also included, i.e. dopamine synapse, glutamateric synapse, nicotine addiction and cAMP signaling pathways, etc. Such a network was a combination of drug addiction and schizophrenia-related networks and illustrated that the overlapped part of two molecular networks were essential in understanding the high nicotine addiction and schizophrenia comorbidity. Of note, the new candidate genes predicted by network analysis can be found in the network which illustrated these new risk factors with high confidence. Moreover, via CaM and PKC many loops were interlinked, further indicating that their important roles in nicotine addiction and schizophrenia. As protein kinases, PKC participates in a wide variety of cellular processes including neurodevelopment. CaM is a multifunctional intermediate calcium-binding messenger protein and acting as part of a calcium signal transduction pathway, CaM also regulates several cellular processes by modifying its interactions with various target proteins such as kinases or phosphatases. Both CaM and PKC are key components for pathways associated to synaptic plasticity and remodeling. Additionally, according to a previous study^68^, the synaptic can rapidly remodel by PKC through reciprocal translocation of NMDA receptors and CaMKII (which is a protein kinase regulated by the Ca^2+^/CaM complex), suggesting that a direct/indirect link between PKC and CaMKII is existent, which may enhance the crosstalk of the pathways in our molecular network.

Nevertheless, there are some limitations in this study. For instance, the pathway-related gene annotation system in KEGG database and PPI network constructed by integrating the published articles may be incomplete due to the limitation of current technology. However, some meaningful and novel interpretation can be found for the association between nicotine addiction and schizophrenia. Moreover, the schizophrenia and nicotine dependence molecular network based on the results of pathway enrichment analysis and network analysis provides a systematic view for understanding the high prevalence tobacco use in schizophrenia.

In summary, we performed a systematic analysis on the susceptive genes related to nicotine addiction and schizophrenia and built the pathogenetic association between two diseases based on pathways enrichment analysis and network analysis. We identified the pathways that may be responsible for the high nicotine addiction and schizophrenia comorbidity. In addition, through network analysis we identified the PPI pairs shared by nicotine addiction and schizophrenia, as well as novel genes potentially linked to the etiology and development of the diseases. Our results may provide an alternative view on exploring the linkage between nicotine addiction and schizophrenia, and suggest that a system level approach used in this work can be promising for understanding the pathogenetic association between diseases.

## Methods

### Data collection

In this study, the susceptibility genes related to nicotine addiction or schizophrenia were collected from three sources including core genes correlated with two diseases, prioritized genes based on mathematic methods, and genes extracted from association study. For nicotine addiction related genes, based on human genetics studies, Li and Burmeister^21^ compiled a list of 49 genes related to nicotine addiction, which were selected as core genes for nicotine dependence. Liu *et al*. obtained 220 genes potentially related to nicotine dependence via a multi-source gene prioritization approach^69^. In addition, 267 genes reported to be positively associated with nicotine addiction in the association studies were also provided by Liu *et al*.^69^. A combination of these three lists resulted in a set of 276 unique genes (denoted as NAgenes, hereafter).

Schizophrenia-related genes were extracted from the following databases: SchiZophrenia Gene Resource^70^ (SZGR: https://bioinfo.uth.edu/SZGR/). This database provides a comprehensive online resource for schizophrenia genetic studies, from which 38 core genes were collected by manual, 218 genes prioritized from multi-dimensional evidences and 278 genes identified from schizophrenia-related association studies were retrieved^71^. A combination of the three lists resulted in a set of 331 unique genes (denoted as SCZgenes, hereafter).

To construct the human protein-protein interaction (PPI) network, we obtained the Protein Interaction Network Analysis (PINA) database^72^ by pooling and curating the unique physical interaction information from six main public protein interaction databases, i.e., BioGRID, IntAct, DIP, MINT, MIPS/MPact, and HPRD. After excluding the redundant and self-interacting pairs, a human PPI network containing 15,093 nodes and 161,419 edges was constructed.

### Gene ontology annotation of gene data

The functional features of NAgenes and SCZgenes were examined to evaluate the relevance of the gene sets to the nicotine addiction or schizophrenia. By means of Gene Ontology (GO) enrichment analysis, the major biological processes that the gene sets involved could be recognized. Here, the online analysis tool DAVID^73^ was used to perform the GO biological processes enrichment analysis and the exported GO terms whose FDR value were less than 0.05 was selected.

### Pathway enrichment analysis

The biochemical pathways enriched in NAgenes and SCZgenes were identified by functional module ClueGO^74^ in Cytoscape^75^. Kyoto Encyclopedia of Genes and Genomes (KEGG) pathway database^76^ was used for pathway enrichment analysis. In ClueGO, the candidate genes were mapped to each of the pathways. Then, a significant value (*p* value) was assigned to measure the significance that the genes of interest participate in a certain pathway based on hypergeometric test. Finally, the corresponding multiple testing correction p-value was calculated with the Benjamini-Hockberg method^77^. Here, the enriched pathways were identified according to the following threshold: ${{\rm{P}}}_{BH}$ < 0.01.

### New candidate genes related to both diseases prediction based on network analysis

We mapped the NAgenes and SCZgenes to human PPI network separately and obtained the NA-specific sub-network and SCZ-specific sub-network which containing relevant seed nodes and the first neighbor nodes (direct interacting nodes with seed nodes). Then we extracted the common PPI pairs in the both sub-network and constructed a new common sub-network for both diseases. Using the “Network Analyzer”^78^ plug-in in Cytoscape, we calculated the degree of the each node in the sub-network. After removing the NAgenes and SCZgenes, the remaining genes could be regarded as new candidate genes for both diseases. We selected the new candidate genes with degrees ≥ 5, i.e., genes directly connected with 5 or more of the shared genes in both nicotine addiction and schizophrenia. The selected genes were regarded as risk factors that may contribute to the pathogenetic association between nicotine addiction and schizophrenia.

## Electronic supplementary material

Supplementary Information
