# Supplementary material for: Analyzing the genes related to nicotine addiction or schizophrenia via a pathway and network based approach
Source: Sci Rep. 2018 Feb 13;8:2894. doi: 10.1038/s41598-018-21297-x (PMC5811491; doi:10.1038/s41598-018-21297-x)
Supplement: Supplementary file 1 — Supplementary Information [file 41598_2018_21297_MOESM1_ESM.doc]

## Supplementary Information

**Analyzing the genes related to nicotine addiction or schizophrenia via a pathway and network based approach**

Ying Hu^1^, Zhonghai Fang^1^, Yichen Yang^1^, Dekai Rohlsen-Neal^2^, Feng Cheng^2*^, and Ju Wang^1*^

^1^ School of Biomedical Engineering, Tianjin Medical University, Tianjin, 300070, China ^2^Department of Pharmaceutical Science, College of Pharmacy, University of South Florida, Tampa, FL 33612, USA

^*^Corresponding authors.

Supplemental Table S1. Candidate genes for nicotine addiction

| **Gene ID** | **Gene Symbol** | **Gene Name** |
| --- | --- | --- |
| 9 | NAT1 | N-acetyltransferase 1 |
| 10 | NAT2 | N-acetyltransferase 2 |
| 52 | ACP1 | acid phosphatase 1, soluble |
| 87 | ACTN1 | actinin alpha 1 |
| 88 | ACTN2 | actinin alpha 2 |
| 125 | ADH1B | alcohol dehydrogenase 1B |
| 150 | ADRA2A | adrenoceptor alpha 2A |
| 154 | ADRB2 | adrenoceptor beta 2 |
| 185 | AGTR1 | angiotensin II receptor type 1 |
| 196 | AHR | aryl hydrocarbon receptor |
| 217 | ALDH2 | aldehyde dehydrogenase 2 family (mitochondrial |
| 322 | APBB1 | amyloid beta precursor protein binding family B member 1 |
| 348 | APOE | apolipoprotein E |
| 387 | RHOA | ras homolog family member A |
| 408 | ARRB1 | arrestin beta 1 |
| 409 | ARRB2 | arrestin beta 2 |
| 595 | CCND1 | cyclin D1 |
| 627 | BDNF | brain derived neurotrophic factor |
| 721 | C4B | complement C4B (Chido blood group) |
| 783 | CACNB2 | calcium voltage-gated channel auxiliary subunit beta 2 |
| 814 | CAMK4 | calcium/calmodulin dependent protein kinase IV |
| 885 | CCK | cholecystokinin |
| 902 | CCNH | cyclin H |
| 929 | CD14 | CD14 molecule |
| 1012 | CDH13 | cadherin 13 |
| 1071 | CETP | cholesteryl ester transfer protein |
| 1103 | CHAT | choline O-acetyltransferase |
| 1124 | CHN2 | chimerin 2 |
| 1128 | CHRM1 | cholinergic receptor muscarinic 1 |
| 1129 | CHRM2 | cholinergic receptor muscarinic 2 |
| 1133 | CHRM5 | cholinergic receptor muscarinic 5 |
| 1134 | CHRNA1 | cholinergic receptor nicotinic alpha 1 subunit |
| 1135 | CHRNA2 | cholinergic receptor nicotinic alpha 2 subunit |
| 1136 | CHRNA3 | cholinergic receptor nicotinic alpha 3 subunit |
| 1137 | CHRNA4 | cholinergic receptor nicotinic alpha 4 subunit |
| 1138 | CHRNA5 | cholinergic receptor nicotinic alpha 5 subunit |
| 1139 | CHRNA7 | cholinergic receptor nicotinic alpha 7 subunit |
| 1140 | CHRNB1 | cholinergic receptor nicotinic beta 1 subunit |
| 1141 | CHRNB2 | cholinergic receptor nicotinic beta 2 subunit |
| 1142 | CHRNB3 | cholinergic receptor nicotinic beta 3 subunit |
| 1143 | CHRNB4 | cholinergic receptor nicotinic beta 4 subunit |
| 1144 | CHRND | cholinergic receptor nicotinic delta subunit |
| 1146 | CHRNG | cholinergic receptor nicotinic gamma subunit |
| 1179 | CLCA1 | chloride channel accessory 1 |
| 1268 | CNR1 | cannabinoid receptor 1 |
| 1312 | COMT | catechol-O-methyltransferase |
| 1385 | CREB1 | cAMP responsive element binding protein 1 |
| 1496 | CTNNA2 | catenin alpha 2 |
| 1535 | CYBA | cytochrome b-245 alpha chain |
| 1543 | CYP1A1 | cytochrome P450 family 1 subfamily A member 1 |
| 1545 | CYP1B1 | cytochrome P450 family 1 subfamily B member 1 |
| 1548 | CYP2A6 | cytochrome P450 family 2 subfamily A member 6 |
| 1555 | CYP2B6 | cytochrome P450 family 2 subfamily B member 6 |
| 1565 | CYP2D6 | cytochrome P450 family 2 subfamily D member 6 |
| 1571 | CYP2E1 | cytochrome P450 family 2 subfamily E member 1 |
| 1586 | CYP17A1 | cytochrome P450 family 17 subfamily A member 1 |
| 1600 | DAB1 | DAB1, reelin adaptor protein |
| 1612 | DAPK1 | death associated protein kinase 1 |
| 1621 | DBH | dopamine beta-hydroxylase |
| 1644 | DDC | dopa decarboxylase |
| 1656 | DDX6 | DEAD-box helicase 6 |
| 1672 | DEFB1 | defensin beta 1 |
| 1728 | NQO1 | NAD(P) H quinone dehydrogenase 1 |
| 1742 | DLG4 | discs large MAGUK scaffold protein 4 |
| 1759 | DNM1 | dynamin 1 |
| 1812 | DRD1 | dopamine receptor D1 |
| 1813 | DRD2 | dopamine receptor D2 |
| 1814 | DRD3 | dopamine receptor D3 |
| 1815 | DRD4 | dopamine receptor D4 |
| 1816 | DRD5 | dopamine receptor D5 |
| 1826 | DSCAM | DS cell adhesion molecule |
| 2052 | EPHX1 | epoxide hydrolase 1 |
| 2053 | EPHX2 | epoxide hydrolase 2 |
| 2068 | ERCC2 | ERCC excision repair 2, TFIIH core complex helicase subunit |
| 2074 | ERCC6 | ERCC excision repair 6, chromatin remodeling factor |
| 2078 | ERG | ERG, ETS transcription factor |
| 2099 | ESR1 | estrogen receptor 1 |
| 2166 | FAAH | fatty acid amide hydrolase |
| 2257 | FGF12 | fibroblast growth factor 12 |
| 2259 | FGF14 | fibroblast growth factor 14 |
| 2326 | FMO1 | flavin containing monooxygenase 1 |
| 2550 | GABBR1 | gamma-aminobutyric acid type B receptor subunit 1 |
| 2555 | GABRA2 | gamma-aminobutyric acid type A receptor alpha2 subunit |
| 2557 | GABRA4 | gamma-aminobutyric acid type A receptor alpha4 subunit |
| 2564 | GABRE | gamma-aminobutyric acid type A receptor epsilon subunit |
| 2587 | GALR1 | galanin receptor 1 |
| 2778 | GNAS | GNAS complex locus |
| 2897 | GRIK1 | glutamate ionotropic receptor kainate type subunit 1 |
| 2898 | GRIK2 | glutamate ionotropic receptor kainate type subunit 2 |
| 2902 | GRIN1 | glutamate ionotropic receptor NMDA type subunit 1 |
| 2903 | GRIN2A | glutamate ionotropic receptor NMDA type subunit 2A |
| 2904 | GRIN2B | glutamate ionotropic receptor NMDA type subunit 2B |
| 2908 | NR3C1 | nuclear receptor subfamily 3 group C member 1 |
| 2912 | GRM2 | glutamate metabotropic receptor 2 |
| 2913 | GRM3 | glutamate metabotropic receptor 3 |
| 2917 | GRM7 | glutamate metabotropic receptor 7 |
| 2944 | GSTM1 | glutathione S-transferase mu 1 |
| 2947 | GSTM3 | glutathione S-transferase mu 3 |
| 2950 | GSTP1 | glutathione S-transferase pi 1 |
| 2952 | GSTT1 | glutathione S-transferase theta 1 |
| 3075 | CFH | complement factor H |
| 3094 | HINT1 | histidine triad nucleotide binding protein 1 |
| 3106 | HLA-B | major histocompatibility complex, class I, B |
| 3117 | HLA-DQA1 | major histocompatibility complex, class II, DQ alpha 1 |
| 3123 | HLA-DRB1 | major histocompatibility complex, class II, DR beta 1 |
| 3240 | HP | haptoglobin |
| 3308 | HSPA4 | heat shock protein family A (Hsp70) member 4 |
| 3355 | HTR1F | 5-hydroxytryptamine receptor 1F |
| 3356 | HTR2A | 5-hydroxytryptamine receptor 2A |
| 3362 | HTR6 | 5-hydroxytryptamine receptor 6 |
| 3383 | ICAM1 | intercellular adhesion molecule 1 |
| 3458 | IFNG | interferon gamma |
| 3569 | IL6 | interleukin 6 |
| 3576 | CXCL8 | C-X-C motif chemokine ligand 8 |
| 3596 | IL13 | interleukin 13 |
| 3600 | IL15 | interleukin 15 |
| 3690 | ITGB3 | integrin subunit beta 3 |
| 3709 | ITPR2 | inositol 1,4,5-trisphosphate receptor type 2 |
| 3763 | KCNJ6 | potassium voltage-gated channel subfamily J member 6 |
| 3776 | KCNK2 | potassium two pore domain channel subfamily K member 2 |
| 3786 | KCNQ3 | potassium voltage-gated channel subfamily Q member 3 |
| 4128 | MAOA | monoamine oxidase A |
| 4129 | MAOB | monoamine oxidase B |
| 4139 | MARK1 | microtubule affinity regulating kinase 1 |
| 4193 | MDM2 | MDM2 proto-oncogene |
| 4216 | MAP3K4 | mitogen-activated protein kinase kinase kinase 4 |
| 4255 | MGMT | O-6-methylguanine-DNA methyltransferase |
| 4292 | MLH1 | mutL homolog 1 |
| 4314 | MMP3 | matrix metallopeptidase 3 |
| 4321 | MMP12 | matrix metallopeptidase 12 |
| 4353 | MPO | myeloperoxidase |
| 4361 | MRE11 | MRE11 homolog, double strand break repair nuclease |
| 4507 | GABAB2 | gamma-aminobutyric acid type B receptor subunit 2 |
| 4524 | MTHFR | methylenetetrahydrofolate reductase |
| 4552 | MTRR | 5-methyltetrahydrofolate-homocysteine methyltransferase reductase |
| 4594 | GRM2 | glutamate metabotropic receptor 2 |
| 4683 | NBN | nibrin |
| 4745 | NELL1 | neural EGFL like 1 |
| 4781 | NFIB | nuclear factor I B |
| 4843 | NOS2 | nitric oxide synthase 2 |
| 4846 | NOS3 | nitric oxide synthase 3 |
| 4852 | NPY | neuropeptide Y |
| 4878 | NPPA | natriuretic peptide A |
| 4886 | NPY1R | neuropeptide Y receptor Y1 |
| 4887 | NPY2R | neuropeptide Y receptor Y2 |
| 4915 | NTRK2 | neurotrophic receptor tyrosine kinase 2 |
| 4929 | NR4A2 | nuclear receptor subfamily 4 group A member 2 |
| 4968 | OGG1 | 8-oxoguanine DNA glycosylase |
| 4985 | OPRD1 | opioid receptor delta 1 |
| 4988 | OPRM1 | opioid receptor mu 1 |
| 5066 | PAM | peptidylglycine alpha-amidating monooxygenase |
| 5071 | PRKN | parkin RBR E3 ubiquitin protein ligase |
| 5137 | PDE1C | phosphodiesterase 1C |
| 5144 | PDE4D | phosphodiesterase 4D |
| 5243 | ABCB1 | ATP binding cassette subfamily B member 1 |
| 5329 | PLAUR | plasminogen activator, urokinase receptor |
| 5444 | PON1 | paraoxonase 1 |
| 5521 | PPP2R2B | protein phosphatase 2 regulatory subunit Bbeta |
| 5591 | PRKDC | protein kinase, DNA-activated, catalytic polypeptide |
| 5592 | PRKG1 | protein kinase, cGMP-dependent, type I |
| 5685 | PSMA4 | proteasome subunit alpha 4 |
| 5726 | TAS2R38 | taste 2 receptor member 38 |
| 5728 | PTEN | phosphatase and tensin homolog |
| 5743 | PTGS2 | prostaglandin-endoperoxide synthase 2 |
| 5789 | PTPRD | protein tyrosine phosphatase, receptor type D |
| 5799 | PTPRN2 | protein tyrosine phosphatase, receptor type N2 |
| 5887 | RAD23B | RAD23 homolog B, nucleotide excision repair protein |
| 6439 | SFTPB | surfactant protein B |
| 6506 | SLC1A2 | solute carrier family 1 member 2 |
| 6531 | SLC6A3 | solute carrier family 6 member 3 |
| 6532 | SLC6A4 | solute carrier family 6 member 4 |
| 6571 | SLC18A2 | solute carrier family 18 member A2 |
| 6648 | SOD2 | superoxide dismutase 2 |
| 6649 | SOD3 | superoxide dismutase 3 |
| 6660 | SOX5 | SRY-box 5 |
| 6817 | SULT1A1 | sulfotransferase family 1A member 1 |
| 7010 | TEK | TEK receptor tyrosine kinase |
| 7040 | TGFB1 | transforming growth factor beta 1 |
| 7054 | TH | tyrosine hydroxylase |
| 7124 | TNF | tumor necrosis factor |
| 7157 | TP53 | tumor protein p53 |
| 7166 | TPH1 | tryptophan hydroxylase 1 |
| 7182 | NR2C2 | nuclear receptor subfamily 2 group C member 2 |
| 7204 | TRIO | trio Rho guanine nucleotide exchange factor |
| 7351 | UCP2 | uncoupling protein 2 |
| 7365 | UGT2B10 | UDP glucuronosyltransferase family 2 member B10 |
| 7399 | USH2A | usherin |
| 7508 | XPC | XPC complex subunit, DNA damage recognition and repair factor |
| 7515 | XRCC1 | X-ray repair cross complementing 1 |
| 7517 | XRCC3 | X-ray repair cross complementing 3 |
| 8464 | SUPT3H | SPT3 homolog, SAGA and STAGA complex component |
| 8973 | CHRNA6 | cholinergic receptor nicotinic alpha 6 subunit |
| 9141 | PDCD5 | programmed cell death 5 |
| 9215 | LARGE1 | LARGE xylosyl- and glucuronyltransferase 1 |
| 9223 | MAGI1 | membrane associated guanylate kinase, WW and PDZ domain containing 1 |
| 9369 | NRXN3 | neurexin 3 |
| 9378 | NRXN1 | neurexin 1 |
| 9403 | SELENOF | selenoprotein F |
| 9455 | HOMER2 | homer scaffolding protein 2 |
| 9456 | HOMER1 | homer scaffolding protein 1 |
| 9467 | SH3BP5 | SH3 domain binding protein 5 |
| 9568 | GABBR2 | gamma-aminobutyric acid type B receptor subunit 2 |
| 9586 | CREB5 | cAMP responsive element binding protein 5 |
| 9844 | ELMO1 | engulfment and cell motility 1 |
| 9904 | RBM19 | RNA binding motif protein 19 |
| 10079 | ATP9A | ATPase phospholipid transporting 9A (putative) |
| 10086 | HHLA1 | HERV-H LTR-associating 1 |
| 10257 | ABCC4 | ATP binding cassette subfamily C member 4 |
| 10411 | RAPGEF3 | Rap guanine nucleotide exchange factor 3 |
| 10512 | SEMA3C | semaphorin 3C |
| 11122 | PTPRT | protein tyrosine phosphatase, receptor type T |
| 11200 | CHEK2 | checkpoint kinase 2 |
| 11214 | AKAP13 | A-kinase anchoring protein 13 |
| 11337 | GABARAP | GABA type A receptor-associated protein |
| 23043 | TNIK | TRAF2 and NCK interacting kinase |
| 23090 | ZNF423 | zinc finger protein 423 |
| 23174 | ZCCHC14 | zinc finger CCHC-type containing 14 |
| 23189 | KANK1 | KN motif and ankyrin repeat domains 1 |
| 23230 | VPS13A | vacuolar protein sorting 13 homolog A |
| 23254 | KAZN | kazrin, periplakin interacting protein |
| 23413 | NCS1 | neuronal calcium sensor 1 |
| 26059 | ERC2 | ELKS/RAB6-interacting/CAST family member 2 |
| 27241 | BBS9 | Bardet-Biedl syndrome 9 |
| 27255 | CNTN6 | contactin 6 |
| 28232 | SLCO3A1 | solute carrier organic anion transporter family member 3A1 |
| 29119 | CTNNA3 | catenin alpha 3 |
| 53358 | SHC3 | SHC adaptor protein 3 |
| 54577 | UGT1A7 | UDP glucuronosyltransferase family 1 member A7 |
| 54715 | RBFOX1 | RNA binding protein, fox-1 homolog 1 |
| 54796 | BNC2 | basonuclin 2 |
| 54970 | TTC12 | tetratricopeptide repeat domain 12 |
| 55214 | P3H2 | prolyl 3-hydroxylase 2 |
| 55691 | FRMD4A | FERM domain containing 4A |
| 55799 | CACNA2D3 | calcium voltage-gated channel auxiliary subunit alpha2delta 3 |
| 56288 | PARD3 | par-3 family cell polarity regulator |
| 57053 | CHRNA10 | cholinergic receptor nicotinic alpha 10 subunit |
| 57113 | TRPC7 | transient receptor potential cation channel subfamily C member 7 |
| 57453 | DSCAML1 | DS cell adhesion molecule like 1 |
| 57509 | MTUS1 | microtubule associated scaffold protein 1 |
| 57568 | SIPA1L2 | signal induced proliferation associated 1 like 2 |
| 57633 | LRRN1 | leucine rich repeat neuronal 1 |
| 59340 | HRH4 | histamine receptor H4 |
| 64084 | CLSTN2 | calsyntenin 2 |
| 64478 | CSMD1 | CUB and Sushi multiple domains 1 |
| 64682 | ANAPC1 | anaphase promoting complex subunit 1 |
| 64754 | SMYD3 | SET and MYND domain containing 3 |
| 64839 | FBXL17 | F-box and leucine rich repeat protein 17 |
| 65217 | PCDH15 | protocadherin related 15 |
| 79068 | FTO | FTO, alpha-ketoglutarate dependent dioxygenase |
| 79658 | ARHGAP10 | Rho GTPase activating protein 10 |
| 79858 | NEK11 | NIMA related kinase 11 |
| 79875 | THSD4 | thrombospondin type 1 domain containing 4 |
| 80333 | KCNIP4 | potassium voltage-gated channel interacting protein 4 |
| 84152 | PPP1R1B | protein phosphatase 1 regulatory inhibitor subunit 1B |
| 84569 | LYZL1 | lysozyme like 1 |
| 84700 | MYO18B | myosin XVIIIB |
| 84953 | MICALCL | MICAL C-terminal like |
| 112398 | EGLN2 | egl-9 family hypoxia inducible factor 2 |
| 114815 | SORCS1 | sortilin related VPS10 domain containing receptor 1 |
| 114876 | OSBPL1A | oxysterol binding protein like 1A |
| 116443 | GRIN3A | glutamate ionotropic receptor NMDA type subunit 3A |
| 121278 | TPH2 | tryptophan hydroxylase 2 |
| 137868 | SGCZ | sarcoglycan zeta |
| 157310 | PEBP4 | phosphatidylethanolamine binding protein 4 |
| 158038 | LINGO2 | leucine rich repeat and Ig domain containing 2 |
| 169792 | GLIS3 | GLIS family zinc finger 3 |
| 255239 | ANKK1 | ankyrin repeat and kinase domain containing 1 |
| 284217 | LAMA1 | laminin subunit alpha 1 |
| 285195 | SLC9A9 | solute carrier family 9 member A9 |
| 387129 | NPSR1 | neuropeptide S receptor 1 |
| 389610 | XKR5 | XK related 5 |
| 400207 | FLJ42220 | FLJ42220 protein |
| 440279 | UNC13C | unc-13 homolog C |
| 442117 | GALNTL6 | polypeptide N-acetylgalactosaminyltransferase-like 6 |
| 493860 | CCDC73 | coiled-coil domain containing 73 |
| 729330 | OC90 | otoconin 90 |

Supplemental Table S2. Candidate genes of schizophrenia

| **Gene ID** | **Gene Symbol** | **Gene Name** |
| --- | --- | --- |
| 116 | ADCYAP1 | adenylate cyclase activating polypeptide 1 |
| 125 | ADH1B | alcohol dehydrogenase 1B (class I), beta polypeptide |
| 148 | ADRA1A | adrenoceptor alpha 1A |
| 207 | AKT1 | AKT serine/threonine kinase 1 |
| 238 | ALK | ALK receptor tyrosine kinase |
| 348 | APOE | apolipoprotein E |
| 355 | FAS | Fas cell surface death receptor |
| 367 | AR | androgen receptor |
| 405 | ARNT | aryl hydrocarbon receptor nuclear translocator |
| 421 | ARVCF | armadillo repeat gene deleted in velocardiofacial syndrome |
| 486 | FXYD2 | FXYD domain containing ion transport regulator 2 |
| 578 | BAK1 | BCL2 antagonist/killer 1 |
| 627 | BDNF | brain derived neurotrophic factor |
| 629 | CFB | complement factor B |
| 688 | KLF5 | Kruppel like factor 5 |
| 718 | C3 | complement C3 |
| 720 | C4A | complement C4A (Rodgers blood group) |
| 721 | C4B | complement C4B (Chido blood group) |
| 753 | LDLRAD4 | low density lipoprotein receptor class A domain containing 4 |
| 778 | CACNA1F | calcium voltage-gated channel subunit alpha1 F |
| 780 | DDR1 | discoidin domain receptor tyrosine kinase 1 |
| 886 | CCKAR | cholecystokinin A receptor |
| 920 | CD4 | CD4 molecule |
| 952 | CD38 | CD38 molecule |
| 1103 | CHAT | choline O-acetyltransferase |
| 1113 | CHGA | chromogranin A |
| 1114 | CHGB | chromogranin B |
| 1116 | CHI3L1 | chitinase 3 like 1 |
| 1124 | CHN2 | chimerin 2 |
| 1133 | CHRM5 | cholinergic receptor muscarinic 5 |
| 1139 | CHRNA7 | cholinergic receptor nicotinic alpha 7 subunit |
| 1141 | CHRNB2 | cholinergic receptor nicotinic beta 2 subunit |
| 1267 | CNP | 2',3'-cyclic nucleotide 3' phosphodiesterase |
| 1268 | CNR1 | cannabinoid receptor 1 |
| 1312 | COMT | catechol-O-methyltransferase |
| 1316 | KLF6 | Kruppel like factor 6 |
| 1388 | ATF6B | activating transcription factor 6 beta |
| 1401 | CRP | C-reactive protein |
| 1438 | CSF2RA | colony stimulating factor 2 receptor alpha subunit |
| 1439 | CSF2RB | colony stimulating factor 2 receptor beta common subunit |
| 1493 | CTLA4 | cytotoxic T-lymphocyte associated protein 4 |
| 1544 | CYP1A2 | cytochrome P450 family 1 subfamily A member 2 |
| 1565 | CYP2D6 | cytochrome P450 family 2 subfamily D member 6 |
| 1610 | DAO | D-amino acid oxidase |
| 1622 | DBI | diazepam binding inhibitor, acyl-CoA binding protein |
| 1674 | DES | desmin |
| 1808 | DPYSL2 | dihydropyrimidinase like 2 |
| 1812 | DRD1 | dopamine receptor D1 |
| 1813 | DRD2 | dopamine receptor D2 |
| 1814 | DRD3 | dopamine receptor D3 |
| 1815 | DRD4 | dopamine receptor D4 |
| 1816 | DRD5 | dopamine receptor D5 |
| 1822 | ATN1 | atrophin 1 |
| 1950 | EGF | epidermal growth factor |
| 1956 | EGFR | epidermal growth factor receptor |
| 1960 | EGR3 | early growth response 3 |
| 2029 | ENSA | endosulfine alpha |
| 2065 | ERBB3 | erb-b2 receptor tyrosine kinase 3 |
| 2066 | ERBB4 | erb-b2 receptor tyrosine kinase 4 |
| 2098 | ESD | esterase D |
| 2161 | F12 | coagulation factor XII |
| 2173 | FABP7 | fatty acid binding protein 7 |
| 2246 | FGF1 | fibroblast growth factor 1 |
| 2259 | FGF14 | fibroblast growth factor 14 |
| 2260 | FGFR1 | fibroblast growth factor receptor 1 |
| 2317 | FLNB | filamin B |
| 2335 | FN1 | fibronectin 1 |
| 2532 | ACKR1 | atypical chemokine receptor 1 (Duffy blood group) |
| 2550 | GABBR1 | gamma-aminobutyric acid type B receptor subunit 1 |
| 2554 | GABRA1 | gamma-aminobutyric acid type A receptor alpha1 subunit |
| 2559 | GABRA6 | gamma-aminobutyric acid type A receptor alpha6 subunit |
| 2561 | GABRB2 | gamma-aminobutyric acid type A receptor beta2 subunit |
| 2566 | GABRG2 | gamma-aminobutyric acid type A receptor gamma2 subunit |
| 2568 | GABRP | gamma-aminobutyric acid type A receptor pi subunit |
| 2571 | GAD1 | glutamate decarboxylase 1 |
| 2638 | GC | GC, vitamin D binding protein |
| 2668 | GDNF | glial cell derived neurotrophic factor |
| 2703 | GJA8 | gap junction protein alpha 8 |
| 2730 | GCLM | glutamate-cysteine ligase modifier subunit |
| 2774 | GNAL | G protein subunit alpha L |
| 2775 | GNAO1 | G protein subunit alpha o1 |
| 2778 | GNAS | GNAS complex locus |
| 2784 | GNB3 | G protein subunit beta 3 |
| 2847 | MCHR1 | melanin concentrating hormone receptor 1 |
| 2890 | GRIA1 | glutamate ionotropic receptor AMPA type subunit 1 |
| 2892 | GRIA3 | glutamate ionotropic receptor AMPA type subunit 3 |
| 2893 | GRIA4 | glutamate ionotropic receptor AMPA type subunit 4 |
| 2894 | GRID1 | glutamate ionotropic receptor delta type subunit 1 |
| 2898 | GRIK2 | glutamate ionotropic receptor kainate type subunit 2 |
| 2899 | GRIK3 | glutamate ionotropic receptor kainate type subunit 3 |
| 2900 | GRIK4 | glutamate ionotropic receptor kainate type subunit 4 |
| 2902 | GRIN1 | glutamate ionotropic receptor NMDA type subunit 1 |
| 2903 | GRIN2A | glutamate ionotropic receptor NMDA type subunit 2A |
| 2904 | GRIN2B | glutamate ionotropic receptor NMDA type subunit 2B |
| 2906 | GRIN2D | glutamate ionotropic receptor NMDA type subunit 2D |
| 2913 | GRM3 | glutamate metabotropic receptor 3 |
| 2914 | GRM4 | glutamate metabotropic receptor 4 |
| 2915 | GRM5 | glutamate metabotropic receptor 5 |
| 2917 | GRM7 | glutamate metabotropic receptor 7 |
| 2918 | GRM8 | glutamate metabotropic receptor 8 |
| 2932 | GSK3B | glycogen synthase kinase 3 beta |
| 2936 | GSR | glutathione-disulfide reductase |
| 2937 | GSS | glutathione synthetase |
| 2944 | GSTM1 | glutathione S-transferase mu 1 |
| 2952 | GSTT1 | glutathione S-transferase theta 1 |
| 3084 | NRG1 | neuregulin 1 |
| 3094 | HINT1 | histidine triad nucleotide binding protein 1 |
| 3145 | HMBS | hydroxymethylbilane synthase |
| 3240 | HP | haptoglobin |
| 3269 | HRH1 | histamine receptor H1 |
| 3274 | HRH2 | histamine receptor H2 |
| 3350 | HTR1A | 5-hydroxytryptamine receptor 1A |
| 3356 | HTR2A | 5-hydroxytryptamine receptor 2A |
| 3358 | HTR2C | 5-hydroxytryptamine receptor 2C |
| 3359 | HTR3A | 5-hydroxytryptamine receptor 3A |
| 3360 | HTR4 | 5-hydroxytryptamine receptor 4 |
| 3361 | HTR5A | 5-hydroxytryptamine receptor 5A |
| 3362 | HTR6 | 5-hydroxytryptamine receptor 6 |
| 3363 | HTR7 | 5-hydroxytryptamine receptor 7 |
| 3552 | IL1A | interleukin 1 alpha |
| 3553 | IL1B | interleukin 1 beta |
| 3557 | IL1RN | interleukin 1 receptor antagonist |
| 3558 | IL2 | interleukin 2 |
| 3562 | IL3 | interleukin 3 |
| 3563 | IL3RA | interleukin 3 receptor subunit alpha |
| 3565 | IL4 | interleukin 4 |
| 3579 | CXCR2 | C-X-C motif chemokine receptor 2 |
| 3586 | IL10 | interleukin 10 |
| 3587 | IL10RA | interleukin 10 receptor subunit alpha |
| 3593 | IL12B | interleukin 12B |
| 3606 | IL18 | interleukin 18 |
| 3613 | IMPA2 | inositol monophosphatase 2 |
| 3623 | INHA | inhibin alpha subunit |
| 3702 | ITK | IL2 inducible T-cell kinase |
| 3720 | JARID2 | jumonji and AT-rich interaction domain containing 2 |
| 3782 | KCNN3 | potassium calcium-activated channel subfamily N member 3 |
| 3796 | KIF2A | kinesin family member 2A |
| 3839 | KPNA3 | karyopherin subunit alpha 3 |
| 3843 | IPO5 | importin 5 |
| 3897 | L1CAM | L1 cell adhesion molecule |
| 4023 | LPL | lipoprotein lipase |
| 4049 | LTA | lymphotoxin alpha |
| 4099 | MAG | myelin associated glycoprotein |
| 4128 | MAOA | monoamine oxidase A |
| 4133 | MAP2 | microtubule associated protein 2 |
| 4135 | MAP6 | microtubule associated protein 6 |
| 4277 | MICB | MHC class I polypeptide-related sequence B |
| 4287 | ATXN3 | ataxin 3 |
| 4332 | MNDA | myeloid cell nuclear differentiation antigen |
| 4340 | MOG | myelin oligodendrocyte glycoprotein |
| 4522 | MTHFD1 | methylenetetrahydrofolate dehydrogenase, cyclohydrolase and formyltetrahydrofolate synthetase 1 |
| 4524 | MTHFR | methylenetetrahydrofolate reductase |
| 4548 | MTR | 5-methyltetrahydrofolate-homocysteine methyltransferase |
| 4650 | MYO9B | myosin IXB |
| 4729 | NDUFV2 | NADH:ubiquinone oxidoreductase core subunit V2 |
| 4762 | NEUROG1 | neurogenin 1 |
| 4835 | NQO2 | N-ribosyldihydronicotinamide:quinone reductase 2 |
| 4842 | NOS1 | nitric oxide synthase 1 |
| 4852 | NPY | neuropeptide Y |
| 4853 | NOTCH2 | notch 2 |
| 4855 | NOTCH4 | notch 4 |
| 4867 | NPHP1 | nephrocystin 1 |
| 4900 | NRGN | neurogranin |
| 4908 | NTF3 | neurotrophin 3 |
| 4929 | NR4A2 | nuclear receptor subfamily 4 group A member 2 |
| 4978 | OPCML | opioid binding protein/cell adhesion molecule like |
| 5066 | PAM | peptidylglycine alpha-amidating monooxygenase |
| 5074 | PAWR | pro-apoptotic WT1 regulator |
| 5080 | PAX6 | paired box 6 |
| 5100 | PCDH8 | protocadherin 8 |
| 5108 | PCM1 | pericentriolar material 1 |
| 5142 | PDE4B | phosphodiesterase 4B |
| 5173 | PDYN | prodynorphin |
| 5243 | ABCB1 | ATP binding cassette subfamily B member 1 |
| 5288 | PIK3C2G | phosphatidylinositol-4-phosphate 3-kinase catalytic subunit type 2 gamma |
| 5289 | PIK3C3 | phosphatidylinositol 3-kinase catalytic subunit type 3 |
| 5297 | PI4KA | phosphatidylinositol 4-kinase alpha |
| 5305 | PIP4K2A | phosphatidylinositol-5-phosphate 4-kinase type 2 alpha |
| 5321 | PLA2G4A | phospholipase A2 group IVA |
| 5354 | PLP1 | proteolipid protein 1 |
| 5362 | PLXNA2 | plexin A2 |
| 5521 | PPP2R2B | protein phosphatase 2 regulatory subunit Bbeta |
| 5533 | PPP3CC | protein phosphatase 3 catalytic subunit gamma |
| 5624 | PROC | protein C, inactivator of coagulation factors Va and VIIIa |
| 5625 | PRODH | proline dehydrogenase 1 |
| 5649 | RELN | reelin |
| 5660 | PSAP | prosaposin |
| 5743 | PTGS2 | prostaglandin-endoperoxide synthase 2 |
| 5803 | PTPRZ1 | protein tyrosine phosphatase, receptor type Z1 |
| 5902 | RANBP1 | RAN binding protein 1 |
| 5947 | RBP1 | retinol binding protein 1 |
| 5999 | RGS4 | regulator of G protein signaling 4 |
| 6271 | S100A1 | S100 calcium binding protein A1 |
| 6281 | S100A10 | S100 calcium binding protein A10 |
| 6285 | S100B | S100 calcium binding protein B |
| 6310 | ATXN1 | ataxin 1 |
| 6422 | SFRP1 | secreted frizzled related protein 1 |
| 6506 | SLC1A2 | solute carrier family 1 member 2 |
| 6511 | SLC1A6 | solute carrier family 1 member 6 |
| 6531 | SLC6A3 | solute carrier family 6 member 3 |
| 6532 | SLC6A4 | solute carrier family 6 member 4 |
| 6570 | SLC18A1 | solute carrier family 18 member A1 |
| 6571 | SLC18A2 | solute carrier family 18 member A2 |
| 6586 | SLIT3 | slit guidance ligand 3 |
| 6648 | SOD2 | superoxide dismutase 2 |
| 6663 | SOX10 | SRY-box 10 |
| 6804 | STX1A | syntaxin 1A |
| 6854 | SYN2 | synapsin II |
| 6899 | TBX1 | T-box 1 |
| 6908 | TBP | TATA-box binding protein |
| 6950 | TCP1 | t-complex 1 |
| 7018 | TF | transferrin |
| 7054 | TH | tyrosine hydroxylase |
| 7078 | TIMP3 | TIMP metallopeptidase inhibitor 3 |
| 7122 | CLDN5 | claudin 5 |
| 7124 | TNF | tumor necrosis factor |
| 7148 | TNXB | tenascin XB |
| 7157 | TP53 | tumor protein p53 |
| 7166 | TPH1 | tryptophan hydroxylase 1 |
| 7257 | TSNAX | translin associated factor X |
| 7351 | UCP2 | uncoupling protein 2 |
| 7353 | UFD1 | ubiquitin recognition factor in ER associated degradation 1 |
| 7494 | XBP1 | X-box binding protein 1 |
| 7515 | XRCC1 | X-ray repair cross complementing 1 |
| 7531 | YWHAE | tyrosine 3-monooxygenase/tryptophan 5-monooxygenase activation protein epsilon |
| 7533 | YWHAH | tyrosine 3-monooxygenase/tryptophan 5-monooxygenase activation protein eta |
| 7534 | YWHAZ | tyrosine 3-monooxygenase/tryptophan 5-monooxygenase activation protein zeta |
| 7851 | MALL | mal, T-cell differentiation protein like |
| 7857 | SCG2 | secretogranin II |
| 7976 | FZD3 | frizzled class receptor 3 |
| 8128 | ST8SIA2 | ST8 alpha-N-acetyl-neuraminide alpha-2,8-sialyltransferase 2 |
| 8214 | DGCR6 | DiGeorge syndrome critical region gene 6 |
| 8220 | DGCR14 | DiGeorge syndrome critical region gene 14 |
| 8224 | SYN3 | synapsin III |
| 8398 | PLA2G6 | phospholipase A2 group VI |
| 8443 | GNPAT | glyceronephosphate O-acyltransferase |
| 8564 | KMO | kynurenine 3-monooxygenase |
| 8605 | PLA2G4C | phospholipase A2 group IVC |
| 8685 | MARCO | macrophage receptor with collagenous structure |
| 8807 | IL18RAP | interleukin 18 receptor accessory protein |
| 8809 | IL18R1 | interleukin 18 receptor 1 |
| 8817 | FGF18 | fibroblast growth factor 18 |
| 8863 | PER3 | period circadian clock 3 |
| 8914 | TIMELESS | timeless circadian clock |
| 8929 | PHOX2B | paired like homeobox 2b |
| 8991 | SELENBP1 | selenium binding protein 1 |
| 9019 | MPZL1 | myelin protein zero like 1 |
| 9145 | SYNGR1 | synaptogyrin 1 |
| 9223 | MAGI1 | membrane associated guanylate kinase, WW and PDZ domain containing 1 |
| 9253 | NUMBL | NUMB like, endocytic adaptor protein |
| 9342 | SNAP29 | synaptosome associated protein 29 |
| 9443 | MED7 | mediator complex subunit 7 |
| 9463 | PICK1 | protein interacting with PRKCA 1 |
| 9481 | SLC25A27 | solute carrier family 25 member 27 |
| 9542 | NRG2 | neuregulin 2 |
| 9575 | CLOCK | clock circadian regulator |
| 9638 | FEZ1 | fasciculation and elongation protein zeta 1 |
| 9639 | ARHGEF10 | Rho guanine nucleotide exchange factor 10 |
| 9685 | CLINT1 | clathrin interactor 1 |
| 9722 | NOS1AP | nitric oxide synthase 1 adaptor protein |
| 9826 | ARHGEF11 | Rho guanine nucleotide exchange factor 11 |
| 9863 | MAGI2 | membrane associated guanylate kinase, WW and PDZ domain containing 2 |
| 9968 | MED12 | mediator complex subunit 12 |
| 9993 | DGCR2 | DiGeorge syndrome critical region gene 2 |
| 10215 | OLIG2 | oligodendrocyte transcription factor 2 |
| 10280 | SIGMAR1 | sigma non-opioid intracellular receptor 1 |
| 10400 | PEMT | phosphatidylethanolamine N-methyltransferase |
| 10611 | PDLIM5 | PDZ and LIM domain 5 |
| 10628 | TXNIP | thioredoxin interacting protein |
| 10712 | FAM189B | family with sequence similarity 189 member B |
| 10718 | NRG3 | neuregulin 3 |
| 10752 | CHL1 | cell adhesion molecule L1 like |
| 10763 | NES | nestin |
| 10814 | CPLX2 | complexin 2 |
| 11178 | LZTS1 | leucine zipper tumor suppressor 1 |
| 22854 | NTNG1 | netrin G1 |
| 23002 | DAAM1 | dishevelled associated activator of morphogenesis 1 |
| 23208 | SYT11 | synaptotagmin 11 |
| 23209 | MLC1 | megalencephalic leukoencephalopathy with subcortical cysts 1 |
| 23305 | ACSL6 | acyl-CoA synthetase long-chain family member 6 |
| 23322 | RPGRIP1L | RPGRIP1 like |
| 23408 | SIRT5 | sirtuin 5 |
| 23500 | DAAM2 | dishevelled associated activator of morphogenesis 2 |
| 23542 | MAPK8IP2 | mitogen-activated protein kinase 8 interacting protein 2 |
| 23543 | RBFOX2 | RNA binding protein, fox-1 homolog 2 |
| 23774 | BRD1 | bromodomain containing 1 |
| 25830 | SULT4A1 | sulfotransferase family 4A member 1 |
| 27037 | TRMT2A | tRNA methyltransferase 2 homolog A |
| 27121 | DKK4 | dickkopf WNT signaling pathway inhibitor 4 |
| 27185 | DISC1 | disrupted in schizophrenia 1 |
| 27201 | GPR78 | G protein-coupled receptor 78 |
| 29801 | ZDHHC8 | zinc finger DHHC-type containing 8 |
| 51141 | INSIG2 | insulin induced gene 2 |
| 51520 | LARS | leucyl-tRNA synthetase |
| 51586 | MED15 | mediator complex subunit 15 |
| 51735 | RAPGEF6 | Rap guanine nucleotide exchange factor 6 |
| 51807 | TUBA8 | tubulin alpha 8 |
| 53826 | FXYD6 | FXYD domain containing ion transport regulator 6 |
| 54456 | MOV10L1 | Mov10 RISC complex RNA helicase like 1 |
| 54584 | GNB1L | G protein subunit beta 1 like |
| 54806 | AHI1 | Abelson helper integration site 1 |
| 55163 | PNPO | pyridoxamine 5'-phosphate oxidase |
| 55806 | HR | HR, lysine demethylase and nuclear receptor corepressor |
| 56990 | CDC42SE2 | CDC42 small effector 2 |
| 57142 | RTN4 | reticulon 4 |
| 63826 | SRR | serine racemase |
| 63827 | BCAN | brevican |
| 63915 | BLOC1S5 | biogenesis of lysosomal organelles complex 1 subunit 5 |
| 64067 | NPAS3 | neuronal PAS domain protein 3 |
| 65078 | RTN4R | reticulon 4 receptor |
| 79137 | RETREG2 | reticulophagy regulator family member 2 |
| 80833 | APOL3 | apolipoprotein L3 |
| 84062 | DTNBP1 | dystrobrevin binding protein 1 |
| 84152 | PPP1R1B | protein phosphatase 1 regulatory inhibitor subunit 1B |
| 84466 | MEGF10 | multiple EGF like domains 10 |
| 84628 | NTNG2 | netrin G2 |
| 89832 | CHRFAM7A | CHRNA7 (exons 5-10) and FAM7A (exons A-E) fusion |
| 91752 | ZNF804A | zinc finger protein 804A |
| 93986 | FOXP2 | forkhead box P2 |
| 126669 | SHE | Src homology 2 domain containing E |
| 127933 | UHMK1 | U2AF homology motif kinase 1 |
| 130749 | CPO | carboxypeptidase O |
| 134265 | AFAP1L1 | actin filament associated protein 1 like 1 |
| 150465 | TTL | tubulin tyrosine ligase |
| 160777 | CCDC60 | coiled-coil domain containing 60 |
| 260425 | MAGI3 | membrane associated guanylate kinase, WW and PDZ domain containing 3 |
| 266553 | OFCC1 | orofacial cleft 1 candidate 1 |
| 267012 | DAOA | D-amino acid oxidase activator |
| 319100 | TAAR6 | trace amine associated receptor 6 |
| 339855 | KY | kyphoscoliosis peptidase |
| 388552 | BLOC1S3 | biogenesis of lysosomal organelles complex 1 subunit 3 |

Supplemental Table S3. Shared genes in both nicotine addiction and schizophrenia

| **Gene ID** | **Gene Symbol** | **Gene Name** |
| --- | --- | --- |
| 125 | ADH1B | alcohol dehydrogenase 1B (class I), beta polypeptide |
| 348 | APOE | apolipoprotein E |
| 627 | BDNF | brain derived neurotrophic factor |
| 721 | C4B | complement C4B (Chido blood group) |
| 1103 | CHAT | choline O-acetyltransferase |
| 1124 | CHN2 | chimerin 2 |
| 1133 | CHRM5 | cholinergic receptor muscarinic 5 |
| 1139 | CHRNA7 | cholinergic receptor nicotinic alpha 7 subunit |
| 1141 | CHRNB2 | cholinergic receptor nicotinic beta 2 subunit |
| 1268 | CNR1 | cannabinoid receptor 1 |
| 1312 | COMT | catechol-O-methyltransferase |
| 1565 | CYP2D6 | cytochrome P450 family 2 subfamily D member 6 |
| 1812 | DRD1 | dopamine receptor D1 |
| 1813 | DRD2 | dopamine receptor D2 |
| 1814 | DRD3 | dopamine receptor D3 |
| 1815 | DRD4 | dopamine receptor D4 |
| 1816 | DRD5 | dopamine receptor D5 |
| 2259 | FGF14 | fibroblast growth factor 14 |
| 2550 | GABBR1 | gamma-aminobutyric acid type B receptor subunit 1 |
| 2778 | GNAS | GNAS complex locus |
| 2898 | GRIK2 | glutamate ionotropic receptor kainate type subunit 2 |
| 2902 | GRIN1 | glutamate ionotropic receptor NMDA type subunit 1 |
| 2903 | GRIN2A | glutamate ionotropic receptor NMDA type subunit 2A |
| 2904 | GRIN2B | glutamate ionotropic receptor NMDA type subunit 2B |
| 2917 | GRM7 | glutamate metabotropic receptor 7 |
| 2944 | GSTM1 | glutathione S-transferase mu 1 |
| 2952 | GSTT1 | glutathione S-transferase theta 1 |
| 3094 | HINT1 | histidine triad nucleotide binding protein 1 |
| 3240 | HP | haptoglobin |
| 3356 | HTR2A | 5-hydroxytryptamine receptor 2A |
| 3362 | HTR6 | 5-hydroxytryptamine receptor 6 |
| 4128 | MAOA | monoamine oxidase A |
| 4524 | MTHFR | methylenetetrahydrofolate reductase |
| 4852 | NPY | neuropeptide Y |
| 4929 | NR4A2 | nuclear receptor subfamily 4 group A member 2 |
| 5066 | PAM | peptidylglycine alpha-amidating monooxygenase |
| 5243 | ABCB1 | ATP binding cassette subfamily B member 1 |
| 5521 | PPP2R2B | protein phosphatase 2 regulatory subunit Bbeta |
| 5743 | PTGS2 | prostaglandin-endoperoxide synthase 2 |
| 6506 | SLC1A2 | solute carrier family 1 member 2 |
| 6531 | SLC6A3 | solute carrier family 6 member 3 |
| 6532 | SLC6A4 | solute carrier family 6 member 4 |
| 6571 | SLC18A2 | solute carrier family 18 member A2 |
| 6648 | SOD2 | superoxide dismutase 2 |
| 7054 | TH | tyrosine hydroxylase |
| 7124 | TNF | tumor necrosis factor |
| 7157 | TP53 | tumor protein p53 |
| 7166 | TPH1 | tryptophan hydroxylase 1 |
| 7351 | UCP2 | uncoupling protein 2 |
| 7515 | XRCC1 | X-ray repair cross complementing 1 |
| 9223 | MAGI1 | membrane associated guanylate kinase, WW and PDZ domain containing 1 |
| 84152 | PPP1R1B | protein phosphatase 1 regulatory inhibitor subunit 1B |

Supplemental Table S4. GO terms enriched in nicotine addiction

| **GO Term ID** | **GO Term Name** | **PValue** | **FDR** | **Genes Included** |
| --- | --- | --- | --- | --- |
| GO:0007268 | synaptic transmission | 3.89E-32 | 6.84E-29 | DRD1, DRD3, GRIK1, DRD2, GRIK2, DRD5, TH, DRD4, SLC6A4, CACNB2, COMT, GABBR2, SLC1A2, KCNQ3, GRIN2B, APOE, CHRNA5, DLG4, CHRNA4, CHRNA6, CHRNA1, SHC3, NQO1, CHAT, HTR1F, CHRNA3, GABRA2, NRXN3, MAOA, GRIN1, GRIN2A, NRXN1, PARK2, DBH, HOMER1, GABARAP, CTNNA2, CAMK4, NPY, CHRM1, GRM7, HTR6, CHRNB4, CHRNB3, CHRNB2, CHRNB1, UNC13C, CHRNA10, HTR2A |
| GO:0019226 | transmission of nerve impulse | 5.63E-32 | 9.90E-29 | GRIK1, GRIK2, SLC6A4, GABBR2, TGFB1, SLC1A2, KCNQ3, GRIN2B, APOE, DLG4, CHRNA5, CHRNA4, CHRNA6, SHC3, CHRNA1, NQO1, HTR1F, CHAT, CHRNA3, NRXN3, GRIN2A, NRXN1, CTNNA2, CHRM5, CAMK4, CHRM1, GRM7, HTR6, UNC13C, DRD1, DRD3, DRD2, DRD5, DRD4, TH, CACNB2, COMT, ERCC2, GABRA2, MAOA, GRIN1, PARK2, DBH, HOMER1, GABARAP, NPY, CHRNB4, CHRNB3, CHRNB2, CHRNB1, CHRNA10, HTR2A |
| GO:0007267 | cell-cell signaling | 4.98E-25 | 8.75E-22 | GRIK1, FGF14, GRIK2, SLC6A4, IL13, FGF12, GABBR2, IL15, SLC1A2, BDNF, KCNQ3, GRIN2B, APOE, CHRNA5, DLG4, CHRNA4, CHRNA6, CHRNA1, SHC3, NQO1, HTR1F, CHAT, CHRNA3, NRXN3, GRIN2A, NRXN1, CTNNA2, CAMK4, CHRM1, GRM7, HTR6, UNC13C, DRD1, DRD3, DRD2, DRD5, DRD4, TH, CACNB2, COMT, TEK, GABRA2, IL6, MAOA, GRIN1, PARK2, DBH, HOMER1, GABARAP, NPY, NTRK2, CHRNB4, CHRNB3, CHRNB2, CHRNB1, CHRNA10, HTR2A |
| GO:0007610 | behavior | 6.40E-25 | 1.13E-21 | OPRM1, PTGS2, GRIK1, GRIK2, SLC6A3, PTEN, SLC1A2, BDNF, GRIN2B, PPP1R1B, IFNG, DLG4, CHRNA5, CHRNA4, CHRNA7, SHC3, CHAT, CHRNA3, DSCAM, GRIN2A, PLAUR, GRM7, DRD1, CCK, DRD3, DRD2, NPY2R, DRD5, TH, DRD4, COMT, CNR1, DEFB1, IL6, IL8, MAOA, GRIN1, NR4A2, NPY1R, PARK2, DBH, SOD2, NPY, NTRK2, CHRNB4, SLC18A2, CHRNB2, CHRNB1, APBB1, OPRD1, HTR2A |
| GO:0050877 | neurological system process | 3.19E-21 | 5.61E-18 | OPRM1, NBN, GRIK1, PTGS2, GRIK2, SLC6A3, SLC6A4, GRIN3A, GABBR2, PTEN, TGFB1, BDNF, SLC1A2, KCNQ3, GRIN2B, APOE, CHRNA5, DLG4, CHRNA4, CHRNA7, CHRNA6, CHRNA1, SHC3, NQO1, USH2A, CHRNA3, HTR1F, CHAT, NRXN3, GRIN2A, NRXN1, CTNNA2, CHRM5, CAMK4, ARRB2, ARRB1, CHRM1, GRM7, HTR6, GNAS, UNC13C, DRD1, CYP1B1, DRD3, DRD2, BBS9, DRD5, DRD4, TH, CACNB2, COMT, ERCC6, PDE1C, ERCC2, GABRA2, MAOA, GRIN1, PCDH15, NPY1R, PARK2, HOMER1, DBH, GABARAP, SOD2, NPY, TAS2R38, CHRNB4, CHRNB3, CHRND, CHRNB2, CHRNB1, CHRNA10, APBB1, HTR2A |
| GO:0014070 | response to organic cyclic substance | 2.68E-16 | 3.89E-13 | DRD1, CYP1A1, DRD3, PTGS2, DRD2, DRD5, SLC6A3, GRIN1, DRD4, EPHX1, COMT, PTEN, TGFB1, CCND1, CHRNB4, ALDH2, CHRNA5, CHRNA4, NOS3, CHRNA7, CHRNB2, CHRNB1, CHRNA3 |
| GO:0031644 | regulation of neurological system process | 3.78E-16 | 5.88E-13 | DRD1, CCK, TNF, GRIK1, DRD3, PTGS2, GRIK2, DRD2, DRD5, DRD4, BDNF, GRIN2B, APOE, IFNG, DLG4, CHRNA3, CHAT, GRIN1, GRIN2A, PARK2, ADRB2, NTRK2, CHRNB4, CHRNB2, HTR2A |
| GO:0030534 | adult behavior | 6.92E-16 | 1.18E-12 | DRD1, GRIK1, DRD3, DRD2, GRIN1, DRD4, NR4A2, PARK2, DBH, SLC1A2, NPY, GRM7, CHRNB4, CHRNA5, CHRNA4, CHRNB2, CHRNB1, CHAT, CHRNA3, OPRD1 |
| GO:0044057 | regulation of system process | 1.01E-15 | 1.75E-12 | DRD1, CCK, TNF, PTGS2, GRIK1, DRD3, GRIK2, DRD2, DRD5, TH, DRD4, AGTR1, BDNF, GRIN2B, APOE, IFNG, DLG4, NOS3, NOS2, CHAT, CHRNA3, GRIN1, GRIN2A, EPHX2, NPY1R, PARK2, DBH, ADRB2, CHRM2, NTRK2, CHRNB4, CHRNB2, HTR2A |
| GO:0010033 | response to organic substance | 2.23E-15 | 3.91E-12 | PTGS2, SLC6A3, GRIN3A, XRCC1, MMP3, PTEN, TGFB1, SLC1A2, GSTM3, GRIN2B, APOE, IFNG, CHRNA5, CHRNA4, NOS3, CHRNA7, CHRNA3, CYP1A1, GRIN2A, ESR1, CCND1, GNAS, DRD1, TNF, CYP1B1, DRD3, DRD2, DRD5, DRD4, TH, PRKDC, NR3C1, COMT, UGT1A7, HSPA4, IL6, CREB1, GRIN1, NR4A2, EPHX1, NPY1R, DBH, CDH13, ALDH2, CHRNB4, SLC18A2, CHRNB2, CHRNB1, CD14 |
| GO:0042417 | dopamine metabolic process | 8.00E-15 | 1.41E-11 | DRD1, DRD3, DRD2, MAOA, SLC6A3, TH, DRD4, NR4A2, GRIN2A, PARK2, COMT, DBH |
| GO:0007611 | learning or memory | 8.17E-15 | 1.44E-11 | DRD1, PTGS2, DRD3, DRD2, DRD5, GRIN1, TH, GRIN2A, COMT, PARK2, DBH, PTEN, BDNF, GRIN2B, GRM7, DLG4, CHRNA7, CHRNB2, SHC3, APBB1, HTR2A |
| GO:0009712 | catechol metabolic process | 1.09E-14 | 1.91E-11 | DDC, DRD1, DRD3, DRD2, MAOA, SLC6A3, DRD4, TH, GRIN2A, NR4A2, COMT, PARK2, DBH, SULT1A1 |
| GO:0006584 | catecholamine metabolic process | 1.09E-14 | 1.91E-11 | DDC, DRD1, DRD3, DRD2, MAOA, SLC6A3, DRD4, TH, GRIN2A, NR4A2, COMT, PARK2, DBH, SULT1A1 |
| GO:0034311 | diol metabolic process | 1.09E-14 | 1.91E-11 | DDC, DRD1, DRD3, DRD2, MAOA, SLC6A3, DRD4, TH, GRIN2A, NR4A2, COMT, PARK2, DBH, SULT1A1 |
| GO:0042493 | response to drug | 1.68E-14 | 2.95E-11 | NBN, DRD1, DRD3, PTGS2, DRD2, SLC6A3, COMT, XRCC1, PTEN, TGFB1, UGT1A7, SLC1A2, BDNF, SEMA3C, NOS3, CHRNA3, GABRE, CYP1A1, CREB1, GRIN2A, TP53, ABCB1, MMP12, SOD2, CCND1, GNAS, HTR2A |
| GO:0018958 | phenol metabolic process | 1.70E-14 | 2.99E-11 | DDC, DRD1, DRD3, DRD2, MAOA, SLC6A3, DRD4, TH, GRIN2A, NR4A2, COMT, PARK2, DBH, SULT1A1 |
| GO:0051969 | regulation of transmission of nerve impulse | 2.02E-14 | 3.55E-11 | DRD1, TNF, GRIK1, PTGS2, DRD3, GRIK2, DRD2, DRD5, GRIN1, DRD4, GRIN2A, PARK2, BDNF, GRIN2B, APOE, NTRK2, IFNG, CHRNB4, DLG4, CHRNB2, CHAT, CHRNA3, HTR2A |
| GO:0050804 | regulation of synaptic transmission | 4.27E-14 | 7.52E-11 | DRD1, TNF, GRIK1, PTGS2, DRD3, DRD2, GRIK2, DRD5, GRIN1, DRD4, GRIN2A, PARK2, BDNF, GRIN2B, APOE, NTRK2, CHRNB4, DLG4, CHRNB2, CHAT, CHRNA3, HTR2A |
| GO:0030808 | regulation of nucleotide biosynthetic process | 8.84E-14 | 1.55E-10 | OPRM1, DRD1, DRD3, DRD2, DRD5, NPY2R, DRD4, GABBR1, NPY1R, GABBR2, CHRM5, ADRB2, GALR1, APOE, GRM7, NTRK2, ADRA2A, NOS3, GNAS, OPRD1 |
| GO:0030802 | regulation of cyclic nucleotide biosynthetic process | 8.84E-14 | 1.55E-10 | OPRM1, DRD1, DRD3, DRD2, DRD5, NPY2R, DRD4, GABBR1, NPY1R, GABBR2, CHRM5, ADRB2, GALR1, APOE, GRM7, NTRK2, ADRA2A, NOS3, GNAS, OPRD1 |
| GO:0019932 | second-messenger-mediated signaling | 1.26E-13 | 2.22E-10 | OPRM1, DRD1, TNF, DRD3, DRD2, NPY2R, DRD5, DRD4, NCS1, AGTR1, GALR1, APOE, CNR1, NOS2, HTR1F, IL8, NPY1R, HOMER1, ADRB2, CHRM5, NPY, CHRM2, CHRM1, HTR6, GNAS, HTR2A, OPRD1 |
| GO:0030799 | regulation of cyclic nucleotide metabolic process | 1.47E-13 | 2.59E-10 | OPRM1, DRD1, DRD3, DRD2, DRD5, NPY2R, DRD4, GABBR1, NPY1R, GABBR2, CHRM5, ADRB2, GALR1, APOE, GRM7, NTRK2, ADRA2A, NOS3, GNAS, OPRD1 |
| GO:0031279 | regulation of cyclase activity | 1.60E-13 | 2.82E-10 | OPRM1, DRD1, DRD3, DRD2, DRD5, NPY2R, DRD4, GABBR1, NPY1R, GABBR2, CHRM5, ADRB2, GALR1, GRM7, NTRK2, ADRA2A, NOS3, GNAS, OPRD1 |
| GO:0051339 | regulation of lyase activity | 2.31E-13 | 4.05E-10 | OPRM1, DRD1, DRD3, DRD2, DRD5, NPY2R, DRD4, GABBR1, NPY1R, GABBR2, CHRM5, ADRB2, GALR1, GRM7, NTRK2, ADRA2A, NOS3, GNAS, OPRD1 |
| GO:0006140 | regulation of nucleotide metabolic process | 2.41E-13 | 4.23E-10 | OPRM1, DRD1, DRD3, DRD2, DRD5, NPY2R, DRD4, GABBR1, NPY1R, GABBR2, CHRM5, ADRB2, GALR1, APOE, GRM7, NTRK2, ADRA2A, NOS3, GNAS, OPRD1 |
| GO:0045471 | response to ethanol | 3.49E-13 | 6.14E-10 | DRD3, DRD2, SLC6A3, GRIN1, DRD4, TH, GRIN2A, GRIN3A, DBH, PTEN, UGT1A7, CCND1, GRIN2B, APOE, NOS3, CHRNB2 |
| GO:0043279 | response to alkaloid | 4.80E-13 | 8.45E-10 | DRD1, DRD3, DRD2, DRD5, SLC6A3, DRD4, GRIN1, CHRNB4, CHRNA5, ALDH2, CHRNA4, CHRNA7, CHRNB2, CHRNB1, CHRNA3 |
| GO:0045761 | regulation of adenylate cyclase activity | 1.22E-12 | 2.14E-09 | OPRM1, DRD1, DRD3, DRD2, DRD5, NPY2R, DRD4, GABBR1, NPY1R, GABBR2, CHRM5, ADRB2, GALR1, GRM7, NTRK2, ADRA2A, GNAS, OPRD1 |
| GO:0006576 | biogenic amine metabolic process | 1.45E-12 | 2.55E-09 | DDC, DRD1, DRD3, DRD2, MAOA, SLC6A3, DRD4, TH, NR4A2, GRIN2A, COMT, PARK2, DBH, SULT1A1, PON1, CETP, TPH1, TPH2 |
| GO:0001964 | startle response | 1.85E-12 | 3.26E-09 | DRD1, GRIN2B, DRD3, DRD2, SLC6A3, GRIN1, GRIN2A, GRIN3A, PARK2, CTNNA2 |
| GO:0051046 | regulation of secretion | 1.92E-12 | 3.38E-09 | IL6, TNF, GRIK1, DRD3, DRD2, CREB1, DRD4, NCS1, IL13, NPY1R, PARK2, TGFB1, AGTR1, GRIN2B, GRM7, IFNG, NTRK2, CHRNB4, CHRNA4, CHRNB2, CHRNA6, NOS2, CHRNA3, HTR2A |
| GO:0019935 | cyclic-nucleotide-mediated signaling | 2.00E-12 | 3.52E-09 | OPRM1, DRD1, DRD3, DRD2, NPY2R, DRD5, DRD4, NPY1R, CHRM5, ADRB2, GALR1, NPY, APOE, CHRM2, CNR1, HTR6, GNAS, NOS2, HTR1F, OPRD1 |
| GO:0007612 | learning | 2.33E-12 | 4.10E-09 | DRD1, DRD3, DRD2, DRD5, GRIN1, TH, GRIN2A, PARK2, COMT, DBH, GRIN2B, GRM7, DLG4, CHRNB2, APBB1 |
| GO:0007187 | G-protein signaling, coupled to cyclic nucleotide second messenger | 2.34E-12 | 4.12E-09 | OPRM1, DRD1, DRD3, DRD2, NPY2R, DRD5, DRD4, NPY1R, CHRM5, ADRB2, GALR1, NPY, CHRM2, CNR1, HTR6, GNAS, NOS2, HTR1F, OPRD1 |
| GO:0014075 | response to amine stimulus | 2.41E-12 | 4.24E-09 | SLC1A2, DRD1, DRD3, DRD2, DRD5, DRD4, GRIN1, NR4A2, GRIN2A, SLC18A2, NOS3, MMP3, DBH |
| GO:0030817 | regulation of cAMP biosynthetic process | 2.88E-12 | 5.06E-09 | OPRM1, DRD1, DRD3, DRD2, DRD5, NPY2R, DRD4, GABBR1, NPY1R, GABBR2, CHRM5, ADRB2, GALR1, GRM7, NTRK2, ADRA2A, GNAS, OPRD1 |
| GO:0042391 | regulation of membrane potential | 3.48E-12 | 6.13E-09 | DRD1, CCK, GRIK1, GRIK2, DRD4, GRIN1, GRIN2A, TGFB1, SOD2, GRIN2B, CHRNB4, CHRNA4, CHRNB2, CHRND, CHRNB1, CHRNA6, CHRNA1, CHRNA3, CHRNG, ERCC2 |
| GO:0030814 | regulation of cAMP metabolic process | 4.00E-12 | 7.04E-09 | OPRM1, DRD1, DRD3, DRD2, DRD5, NPY2R, DRD4, GABBR1, NPY1R, GABBR2, CHRM5, ADRB2, GALR1, GRM7, NTRK2, ADRA2A, GNAS, OPRD1 |
| GO:0007626 | locomotory behavior | 4.55E-12 | 8.00E-09 | OPRM1, DRD1, DRD3, DRD2, NPY2R, SLC6A3, DRD4, TH, IFNG, CHRNA4, DEFB1, CHAT, CHRNA3, DSCAM, IL6, IL8, GRIN1, NR4A2, PARK2, NPY1R, DBH, SOD2, PLAUR, CHRNB4, SLC18A2, CHRNB2, OPRD1 |
| GO:0010243 | response to organic nitrogen | 4.79E-12 | 8.42E-09 | DRD1, DRD3, PTGS2, DRD2, DRD5, GRIN1, DRD4, NR4A2, GRIN2A, MMP3, DBH, CCND1, SLC1A2, SLC18A2, NOS3 |
| GO:0001975 | response to amphetamine | 1.43E-11 | 2.51E-08 | DRD1, DRD3, DRD2, DRD5, DRD4, GRIN1, NR4A2, GRIN2A, SLC18A2, DBH |
| GO:0006575 | cellular amino acid derivative metabolic process | 2.00E-11 | 3.51E-08 | DDC, DRD1, CYP1A1, DRD3, DRD2, MAOA, SLC6A3, TH, DRD4, NR4A2, GRIN2A, GSTT1, COMT, PARK2, DBH, SOD2, SULT1A1, PON1, CETP, TPH1, TPH2 |
| GO:0007271 | synaptic transmission, cholinergic | 2.82E-11 | 4.97E-08 | APOE, CHRNB4, CHRNB3, CHRNA4, CHRNB2, CHRNB1, NQO1, CHRNA10, CHRNA3 |
| GO:0006873 | cellular ion homeostasis | 3.72E-11 | 6.54E-08 | DRD1, CCK, GRIK1, DRD3, GRIK2, DRD2, DRD5, DRD4, HP, TGFB1, AGTR1, GRIN2B, APOE, CHRNA4, CHRNA7, CHRNA6, CHRNA1, CHRNA3, ERCC2, GRIN1, GRIN2A, EPHX2, TP53, NPY1R, SOD2, CHRNB4, CHRND, CHRNB2, CHRNB1, CHRNG |
| GO:0042592 | homeostatic process | 4.98E-11 | 8.75E-08 | SLC9A9, PARD3, DRD1, NBN, CCK, DRD3, GRIK1, DRD2, GRIK2, MRE11A, DRD5, DRD4, PRKDC, EGLN2, HP, TGFB1, AGTR1, GRIN2B, APOE, IFNG, CHRNA4, CHRNA7, CETP, CHRNA6, CHRNA1, CHRNA3, ERCC2, IL6, GRIN1, TP53, GRIN2A, EPHX2, NPY1R, DBH, SOD2, ADRB2, LARGE, CHRNB4, CHRND, CHRNB2, CHRNB1, CHRNG, HTR2A |
| GO:0055082 | cellular chemical homeostasis | 5.45E-11 | 9.59E-08 | DRD1, CCK, GRIK1, DRD3, GRIK2, DRD2, DRD5, DRD4, HP, TGFB1, AGTR1, GRIN2B, APOE, CHRNA4, CHRNA7, CHRNA6, CHRNA1, CHRNA3, ERCC2, GRIN1, GRIN2A, EPHX2, TP53, NPY1R, SOD2, CHRNB4, CHRND, CHRNB2, CHRNB1, CHRNG |
| GO:0007613 | memory | 5.89E-11 | 1.04E-07 | DRD1, PTGS2, GRIN2B, GRM7, TH, GRIN1, GRIN2A, CHRNA7, CHRNB2, DBH, PTEN, HTR2A |
| GO:0050905 | neuromuscular process | 6.20E-11 | 1.09E-07 | NBN, DRD1, DRD3, DRD2, SLC6A3, GRIN1, GRIN2A, PCDH15, GRIN3A, PARK2, CTNNA2, GRIN2B, CHRND, CHRNA1 |
| GO:0050801 | ion homeostasis | 6.62E-11 | 1.16E-07 | SLC9A9, DRD1, CCK, GRIK1, DRD3, GRIK2, DRD2, DRD5, DRD4, HP, TGFB1, AGTR1, GRIN2B, APOE, CHRNA4, CHRNA7, CHRNA6, CHRNA1, CHRNA3, ERCC2, GRIN1, GRIN2A, EPHX2, TP53, NPY1R, SOD2, CHRNB4, CHRND, CHRNB2, CHRNB1, CHRNG |
| GO:0044093 | positive regulation of molecular function | 1.10E-10 | 1.93E-07 | OPRM1, PARD3, DRD1, CCK, TNF, DRD3, DRD2, DRD5, DRD4, TGFB1, AGTR1, ERCC6, DAB1, MAP3K4, GALR1, APOE, IFNG, ADRA2A, NOS3, CHRNA7, CHRNA3, ERCC2, ANAPC1, ICAM1, IL6, NR4A2, TP53, HOMER1, ADRB2, CCND1, CHRM2, CHRM1, PSMA4, NTRK2, PON1, GNAS, HTR2A |
| GO:0051952 | regulation of amine transport | 2.03E-10 | 3.57E-07 | DRD1, TNF, DRD3, GRIK1, DRD2, DRD4, CHRNA4, CHRNB2, CHRNA6, CHRNA3, HTR2A |
| GO:0007194 | negative regulation of adenylate cyclase activity | 2.10E-10 | 3.69E-07 | OPRM1, CHRM5, GALR1, DRD3, DRD2, NPY2R, GRM7, DRD4, GABBR1, ADRA2A, NPY1R, GABBR2, OPRD1 |
| GO:0051350 | negative regulation of lyase activity | 2.10E-10 | 3.69E-07 | OPRM1, CHRM5, GALR1, DRD3, DRD2, NPY2R, GRM7, DRD4, GABBR1, ADRA2A, NPY1R, GABBR2, OPRD1 |
| GO:0031280 | negative regulation of cyclase activity | 2.10E-10 | 3.69E-07 | OPRM1, CHRM5, GALR1, DRD3, DRD2, NPY2R, GRM7, DRD4, GABBR1, ADRA2A, NPY1R, GABBR2, OPRD1 |
| GO:0035094 | response to nicotine | 2.18E-10 | 3.84E-07 | SLC6A3, CHRNA5, CHRNB4, ALDH2, CHRNA4, CHRNA7, CHRNB2, CHRNB1, CHRNA3 |
| GO:0019725 | cellular homeostasis | 3.57E-10 | 6.28E-07 | DRD1, CCK, GRIK1, DRD3, GRIK2, DRD2, DRD5, DRD4, EGLN2, HP, TGFB1, AGTR1, GRIN2B, APOE, CHRNA4, CHRNA7, CHRNA6, CHRNA1, CHRNA3, ERCC2, GRIN1, GRIN2A, EPHX2, TP53, NPY1R, SOD2, LARGE, CHRNB4, CHRND, CHRNB2, CHRNB1, CHRNG |
| GO:0032101 | regulation of response to external stimulus | 5.89E-10 | 1.04E-06 | DRD1, IL6, CCK, DRD3, IL8, PTGS2, DRD2, SLC6A3, GRIN1, GRIN3A, TGFB1, CTNNA2, AGTR1, CDH13, ADRB2, NPY, APOE, NOS3, CHRNA7 |
| GO:0042596 | fear response | 6.57E-10 | 1.16E-06 | DRD1, BDNF, CCK, GRIN2B, GRIK2, GRM7, DRD4, ADRA2A, DBH |
| GO:0060341 | regulation of cellular localization | 7.97E-10 | 1.40E-06 | IL6, TNF, DRD3, DRD2, CREB1, DRD4, NCS1, IL13, NPY1R, PARK2, TGFB1, GRIN2B, GRM7, IFNG, NTRK2, RHOA, CHRNB4, CHRNA4, CHRNB2, CHRNA6, NOS2, CHRNA3, HTR2A |
| GO:0048878 | chemical homeostasis | 8.70E-10 | 1.53E-06 | SLC9A9, DRD1, CCK, GRIK1, DRD3, GRIK2, DRD2, DRD5, DRD4, HP, TGFB1, AGTR1, GRIN2B, APOE, CHRNA4, CHRNA7, CETP, CHRNA6, CHRNA1, CHRNA3, ERCC2, GRIN1, GRIN2A, EPHX2, TP53, NPY1R, DBH, SOD2, CHRNB4, CHRND, CHRNB2, CHRNB1, CHRNG |
| GO:0007242 | intracellular signaling cascade | 1.21E-09 | 2.13E-06 | OPRM1, NBN, NCS1, MLH1, AGTR1, DAB1, MAP3K4, GALR1, APOE, IFNG, RHOA, CHRNA7, RAPGEF3, NOS2, SHC3, HTR1F, TNIK, TP53, ESR1, NEK11, MARK1, ELMO1, DAPK1, CCND1, ADRB2, CHRM5, XPC, CHRM2, CHRM1, HTR6, GNAS, UNC13C, DRD1, TNF, DRD3, DRD2, NPY2R, DRD5, DRD4, AKAP13, EGLN2, NR3C1, CHEK2, ERCC6, CNR1, ADRA2A, IL8, NPY1R, HOMER1, SH3BP5, CDH13, NPY, CHN2, OPRD1, HTR2A |
| GO:0033555 | multicellular organismal response to stress | 1.27E-09 | 2.23E-06 | DRD1, BDNF, CCK, GRIN2B, GRIK1, GRIK2, GRM7, DRD4, ADRA2A, COMT, DBH |
| GO:0043085 | positive regulation of catalytic activity | 1.28E-09 | 2.25E-06 | OPRM1, PARD3, DRD1, CCK, TNF, DRD3, DRD2, DRD5, DRD4, TGFB1, AGTR1, ERCC6, DAB1, MAP3K4, GALR1, APOE, IFNG, ADRA2A, NOS3, CHRNA7, CHRNA3, ANAPC1, NR4A2, TP53, HOMER1, ADRB2, CCND1, CHRM2, PSMA4, CHRM1, NTRK2, GNAS, HTR2A |
| GO:0007632 | visual behavior | 2.58E-09 | 4.53E-06 | SLC1A2, DRD1, DRD3, DRD2, DRD5, GRIN1, GRIN2A, CHRNB2, DBH, APBB1 |
| GO:0007188 | G-protein signaling, coupled to cAMP nucleotide second messenger | 3.81E-09 | 6.71E-06 | OPRM1, ADRB2, CHRM5, DRD1, GALR1, DRD3, CHRM2, DRD2, NPY2R, DRD5, DRD4, GNAS, NPY1R, OPRD1 |
| GO:0009719 | response to endogenous stimulus | 5.15E-09 | 9.05E-06 | DRD1, TNF, DRD3, PTGS2, DRD2, DRD5, DRD4, TH, PRKDC, MMP3, PTEN, TGFB1, UGT1A7, SLC1A2, GSTM3, NOS3, IL6, GRIN1, GRIN2A, NR4A2, ESR1, NPY1R, DBH, CDH13, CCND1, ALDH2, SLC18A2, GNAS |
| GO:0060134 | prepulse inhibition | 6.56E-09 | 1.15E-05 | DRD1, DRD3, DRD2, SLC6A3, GRIN1, GRIN3A, CTNNA2 |
| GO:0006928 | cell motion | 9.69E-09 | 1.70E-05 | DRD1, CCK, TNF, PTGS2, DRD2, PRKDC, IL13, PRKG1, PTEN, TGFB1, BDNF, DAB1, IFNG, ADRA2A, SEMA3C, NOS3, ICAM1, IL6, IL8, NRXN3, NR4A2, NRXN1, DBH, ELMO1, CTNNA2, PLAUR, CDH13, LAMA1, NPY, APBB1 |
| GO:0008306 | associative learning | 9.78E-09 | 1.72E-05 | DRD1, GRIN2B, DRD2, GRM7, DRD5, GRIN1, CHRNB2, DBH |
| GO:0006811 | ion transport | 1.40E-08 | 2.46E-05 | SLC9A9, GRIK1, DRD2, GRIK2, CACNB2, GRIN3A, KCNIP4, SLC1A2, KCNQ3, GRIN2B, CHRNA5, CHRNA4, CHRNA7, SLCO3A1, CHRNA6, CHRNA1, CHRNA3, CHRNA2, GABRE, GABRA2, CLCA1, GABRA4, TRPC7, GRIN1, GRIN2A, CACNA2D3, KCNK2, ITPR2, KCNJ6, NPY, UCP2, CHRNB4, CHRNB3, ABCC4, CHRND, CHRNB2, CHRNB1, CHRNA10, CHRNG |
| GO:0042981 | regulation of apoptosis | 1.51E-08 | 2.66E-05 | CCK, TNF, PTGS2, GRIK2, NELL1, MGMT, PRKDC, AKAP13, MLH1, CHEK2, NR3C1, PTEN, TGFB1, BDNF, ERCC6, APOE, IFNG, RHOA, NOS3, NQO1, ERCC2, IL6, CREB1, GRIN1, ESR1, GRIN2A, NR4A2, TP53, TRIO, ACTN1, ACTN2, DBH, DAPK1, SOD2, CDH13, ADRB2, MPO, PDCD5, APBB1, GSTP1 |
| GO:0019933 | cAMP-mediated signaling | 1.53E-08 | 2.70E-05 | OPRM1, ADRB2, CHRM5, DRD1, GALR1, DRD3, CHRM2, DRD2, NPY2R, DRD5, DRD4, GNAS, NPY1R, OPRD1 |
| GO:0008542 | visual learning | 1.70E-08 | 2.99E-05 | DRD1, DRD3, DRD2, DRD5, GRIN1, GRIN2A, CHRNB2, DBH, APBB1 |
| GO:0032102 | negative regulation of response to external stimulus | 1.72E-08 | 3.02E-05 | ADRB2, DRD1, CCK, DRD3, DRD2, APOE, SLC6A3, GRIN1, CHRNA7, GRIN3A, CTNNA2 |
| GO:0043067 | regulation of programmed cell death | 1.98E-08 | 3.49E-05 | CCK, TNF, PTGS2, GRIK2, NELL1, MGMT, PRKDC, AKAP13, MLH1, CHEK2, NR3C1, PTEN, TGFB1, BDNF, ERCC6, APOE, IFNG, RHOA, NOS3, NQO1, ERCC2, IL6, CREB1, GRIN1, ESR1, GRIN2A, NR4A2, TP53, TRIO, ACTN1, ACTN2, DBH, DAPK1, SOD2, CDH13, ADRB2, MPO, PDCD5, APBB1, GSTP1 |
| GO:0010941 | regulation of cell death | 2.19E-08 | 3.86E-05 | CCK, TNF, PTGS2, GRIK2, NELL1, MGMT, PRKDC, AKAP13, MLH1, CHEK2, NR3C1, PTEN, TGFB1, BDNF, ERCC6, APOE, IFNG, RHOA, NOS3, NQO1, ERCC2, IL6, CREB1, GRIN1, ESR1, GRIN2A, NR4A2, TP53, TRIO, ACTN1, ACTN2, DBH, DAPK1, SOD2, CDH13, ADRB2, MPO, PDCD5, APBB1, GSTP1 |
| GO:0009314 | response to radiation | 2.33E-08 | 4.11E-05 | DRD1, DRD3, DRD2, DRD5, GRIN1, GRIN2A, TP53, PRKDC, DBH, TGFB1, SOD2, CCND1, SLC1A2, ERCC6, XPC, ARRB1, CHRNB2, APBB1, ERCC2 |
| GO:0008217 | regulation of blood pressure | 2.89E-08 | 5.07E-05 | AGTR1, CYBA, ADRB2, PTGS2, NPY, DRD3, DRD2, DRD5, EPHX2, NOS3, NPY1R, NOS2, NPPA, SOD2 |
| GO:0009416 | response to light stimulus | 2.90E-08 | 5.10E-05 | DRD1, DRD3, DRD2, DRD5, GRIN1, TP53, GRIN2A, DBH, CCND1, SLC1A2, ERCC6, XPC, ARRB1, CHRNB2, APBB1, ERCC2 |
| GO:0043086 | negative regulation of catalytic activity | 3.20E-08 | 5.62E-05 | OPRM1, ANAPC1, DRD1, DRD3, DRD2, NPY2R, DRD5, DRD4, GABBR1, TP53, NPY1R, GABBR2, SH3BP5, CHRM5, GALR1, APOE, GRM7, PSMA4, ADRA2A, NOS3, NQO1, OPRD1 |
| GO:0035095 | behavioral response to nicotine | 3.81E-08 | 6.71E-05 | CHRNA5, CHRNB4, CHRNA4, CHRNB2, CHRNB1, CHRNA3 |
| GO:0007631 | feeding behavior | 3.97E-08 | 6.98E-05 | DRD1, BDNF, CCK, NPY, GRIN2B, DRD2, GRM7, TH, GRIN1, NTRK2, CHRNB2, NPY1R |
| GO:0048169 | regulation of long-term neuronal synaptic plasticity | 5.49E-08 | 9.66E-05 | DRD1, GRIN2B, GRIK2, DRD2, DRD5, GRIN1, DLG4, GRIN2A |
| GO:0007270 | nerve-nerve synaptic transmission | 5.68E-08 | 9.99E-05 | DRD1, CAMK4, DRD3, DRD2, DRD5, TH, GRIN1, PARK2, SHC3 |
| GO:0009628 | response to abiotic stimulus | 5.83E-08 | 1.02E-04 | DRD1, TNF, DRD3, DRD2, DRD5, PRKDC, TGFB1, SLC1A2, ERCC6, GRIN2B, HSPA4, NOS3, ERCC2, GRIN1, TP53, GRIN2A, DBH, SOD2, CCND1, ADRB2, XPC, ARRB1, CHRNB2, CHRNA10, APBB1 |
| GO:0051899 | membrane depolarization | 5.97E-08 | 1.05E-04 | CCK, GRIN2B, GRIK1, GRIK2, GRIN1, GRIN2A, CHRNA4, CHRNB2, CHRNA6, CHRNA3 |
| GO:0007166 | cell surface receptor linked signal transduction | 6.54E-08 | 1.15E-04 | OPRM1, GRIK1, GRIK2, GABBR1, GRIN3A, GABBR2, PTEN, TGFB1, AGTR1, GALR1, GRIN2B, APOE, IFNG, NOS3, NOS2, NPSR1, SHC3, CHRNA3, HTR1F, MAGI1, CNTN6, GRIN2A, PTPRT, CHRM5, CCND1, ADRB2, ARRB2, CHRM2, CHRM1, GRM7, HTR6, GNAS, DRD1, PARD3, CCK, DRD3, DRD2, NPY2R, DRD5, DRD4, SORCS1, ITGB3, CNR1, TEK, HRH4, ADRA2A, DEFB1, GABRE, PTPRD, GABRA2, GABRA4, IL8, CREB1, GRIN1, TRIO, NPY1R, HOMER1, KCNK2, HOMER2, LAMA1, NPY, TAS2R38, NTRK2, CD14, HTR2A, OPRD1 |
| GO:0051240 | positive regulation of multicellular organismal process | 9.70E-08 | 1.71E-04 | IL6, DRD1, TNF, GRIK1, PTGS2, DRD2, GRIK2, CREB1, SLC6A3, EPHX2, NPY1R, DBH, TGFB1, ADRB2, IFNG, NTRK2, NOS3, CHRNB2, CD14, HTR2A |
| GO:0042053 | regulation of dopamine metabolic process | 1.00E-07 | 1.76E-04 | DRD1, SLC6A3, DRD4, NR4A2, CHRNB2, COMT |
| GO:0043271 | negative regulation of ion transport | 1.12E-07 | 1.97E-04 | ADRB2, TNF, PTGS2, DRD2, DRD4, NOS3, TGFB1, HTR2A |
| GO:0060078 | regulation of postsynaptic membrane potential | 1.12E-07 | 1.97E-04 | GRIN2B, GRIK1, GRIK2, DRD4, GRIN1, GRIN2A, CHRNA4, CHRNA3 |
| GO:0051050 | positive regulation of transport | 1.24E-07 | 2.18E-04 | IL6, DRD1, TNF, GRIK1, DRD2, CREB1, DRD4, NCS1, IL13, HOMER1, TGFB1, SLC1A2, ADRB2, GRIN2B, APOE, IFNG, RHOA, PON1, CHRNB2 |

Supplemental Table S5. GO terms enriched in schizophrenia

| **GO Term ID** | **GO Term Name** | **PValue** | **FDR** | **Genes included** |
| --- | --- | --- | --- | --- |
| GO:0007268 | synaptic transmission | 2.48E-30 | 4.40E-27 | GRIK2, GABRB2, SLC6A4, GRIK4, CNP, SLC1A2, GRIN2B, APOE, SLC1A6, GRIN2D, CHAT, GABRG2, EGR3, STX1A, GRIN2A, PI4KA, PDYN, GRM5, GRM4, GRM3, GRM8, HTR7, GRM7, HTR6, DRD1, DRD3, DRD2, DRD5, TH, DRD4, COMT, HRH1, SYN3, SYN2, HTR3A, GAD1, PLP1, NOS1, NTF3, GABRA6, MAOA, GRIN1, GRIA4, ATXN1, ATXN3, NPY, GRIA1, KCNN3, CHRNB2, HTR2C, HTR2A |
| GO:0007267 | cell-cell signaling | 1.77E-30 | 3.15E-27 | FGF18, MPZL1, GABRB2, FGF14, GRIK2, IL18, SLC6A4, GRIK4, CNP, GDNF, IL10, BDNF, SLC1A2, GRIN2B, APOE, GRIN2D, SLC1A6, IL1B, FGF1, LTA, CHAT, GABRG2, STX1A, AR, EGR3, GRIN2A, PI4KA, PCDH8, INHA, PDYN, GRM5, GRM4, GRM3, GRM8, GRM7, HTR7, HTR6, DRD1, YWHAZ, DRD3, DRD2, DRD5, DRD4, TH, COMT, ADCYAP1, HRH1, SYN3, SYN2, HTR3A, GAD1, IL3, PLP1, NOS1, NTF3, MAOA, GABRA6, GRIN1, GRIA4, ATXN1, ATXN3, NPY, GRIA1, KCNN3, ADRA1A, CHRNB2, HTR2C, HTR2A, IL2 |
| GO:0019226 | transmission of nerve impulse | 6.97E-29 | 1.24E-25 | GABRB2, GRIK2, SLC6A4, GRIK4, CNP, SLC1A2, GRIN2B, APOE, SLC1A6, GRIN2D, OLIG2, CHAT, GABRG2, EGR3, STX1A, GRIN2A, PI4KA, PDYN, GRM5, GRM4, GRM3, CHRM5, GRM8, HTR7, GRM7, HTR6, DRD1, DRD3, DRD2, DRD5, TH, DRD4, COMT, HRH1, SYN3, SYN2, HTR3A, GAD1, PLP1, NOS1, NTF3, MAOA, GABRA6, GRIN1, GRIA4, ATXN1, ATXN3, NPY, GRIA1, KCNN3, CHRNB2, HTR2C, HTR2A |
| GO:0031644 | regulation of neurological system process | 5.53E-25 | 9.81E-22 | TF, DRD1, LZTS1, TNF, ERBB4, PTGS2, DRD3, GRIK2, DRD2, GRIK3, DRD5, DRD4, BCAN, SYNGR1, GDNF, IL10, BDNF, GRIN2B, APOE, GRIN2D, CHAT, LTA, EGFR, STX1A, NTF3, GRIN1, GRIN2A, GRIA4, CD38, GRM3, YWHAH, GRM8, CHRNB2, HTR2C, HTR2A |
| GO:0050804 | regulation of synaptic transmission | 3.25E-23 | 5.76E-20 | DRD1, TNF, LZTS1, ERBB4, PTGS2, DRD3, GRIK2, DRD2, GRIK3, DRD5, DRD4, BCAN, SYNGR1, GDNF, BDNF, GRIN2B, APOE, CHAT, LTA, EGFR, STX1A, NTF3, GRIN1, GRIN2A, GRIA4, CD38, GRM3, YWHAH, GRM8, CHRNB2, HTR2C, HTR2A |
| GO:0051969 | regulation of transmission of nerve impulse | 2.86E-23 | 5.08E-20 | TF, DRD1, LZTS1, TNF, ERBB4, PTGS2, DRD3, GRIK2, DRD2, GRIK3, DRD5, DRD4, BCAN, SYNGR1, GDNF, BDNF, GRIN2B, APOE, CHAT, LTA, EGFR, STX1A, NTF3, GRIN1, GRIN2A, GRIA4, CD38, GRM3, YWHAH, GRM8, CHRNB2, HTR2C, HTR2A |
| GO:0044057 | regulation of system process | 7.26E-22 | 1.29E-18 | TF, DRD1, LZTS1, TNF, ERBB4, PTGS2, DRD3, GRIK2, DRD2, GRIK3, DRD5, TH, DRD4, BCAN, SYNGR1, GDNF, IL10, BDNF, DES, GRIN2B, APOE, GRIN2D, IL1B, CHAT, S100A1, LTA, EGFR, STX1A, GNAO1, NOS1, NTF3, GRIN1, GRIN2A, GRIA4, INHA, CD38, GRM3, YWHAH, GRM8, CHRNB2, HTR2C, HTR2A, IL2 |
| GO:0007610 | behavior | 3.65E-21 | 6.47E-18 | MCHR1, CCKAR, PTGS2, GRIK2, IL18, SLC6A3, CNP, GDNF, IL10, SLC1A2, BDNF, HTR1A, GRIN2B, PPP1R1B, GRIN2D, IL1B, CHRNA7, CHRFAM7A, CHAT, GABRG2, GNB1L, GRIN2A, GRM4, GRM7, RELN, DRD1, DRD3, DRD2, DRD5, TH, DRD4, COMT, NPAS3, CNR1, NPHP1, SCG2, IL4, GNAO1, MAOA, GRIN1, NR4A2, TBX1, FOXP2, SOD2, ATXN1, NPY, S100B, GRIA1, SLC18A2, CHRNB2, HTR2C, HTR2A |
| GO:0050877 | neurological system process | 1.46E-14 | 2.60E-11 | GJA8, PTGS2, GABRB2, GRIK2, SLC6A3, SLC6A4, GRIK4, PAX6, CNP, BDNF, SLC1A2, GRIN2B, APOE, GRIN2D, SLC1A6, IL1B, CHRNA7, OLIG2, CHRFAM7A, CHAT, GABRG2, STX1A, EGR3, GRIN2A, PI4KA, PDYN, GRM5, GNAL, GRM4, GRM3, CHRM5, GRM8, GRM7, HTR7, HTR6, GNAS, IL12B, DRD1, DRD3, DRD2, DRD5, DRD4, TH, COMT, TIMP3, DTNBP1, HRH1, SYN3, SYN2, HTR3A, GAD1, PLP1, NOS1, NTF3, MAOA, GABRA6, GRIN1, TBX1, GRIA4, SOD2, FOXP2, ATXN1, ATXN3, S100B, NPY, GRIA1, KCNN3, CHRNB2, CACNA1F, FABP7, HTR2C, HTR2A |
| GO:0042493 | response to drug | 2.65E-14 | 4.70E-11 | YWHAZ, DRD1, ERBB4, DRD3, PTGS2, ERBB3, DRD2, SLC6A3, COMT, XRCC1, GCLM, DGCR2, SLC1A2, BDNF, BLOC1S3, SRR, PEMT, IL1B, TXNIP, NES, GNAO1, GRIN2A, TP53, ABCB1, SOD2, GNAS, SLC18A1, HTR2C, HTR2A |
| GO:0019932 | second-messenger-mediated signaling | 3.28E-14 | 5.83E-11 | CCKAR, MCHR1, DRD1, TNF, DRD3, DRD2, DRD5, DRD4, HRH1, HTR1A, HRH2, APOE, CNR1, PIK3C3, HTR5A, LTA, EGFR, PIK3C2G, PI4KA, HTR4, GRM5, GNAL, GRM4, CHRM5, NPY, HTR7, HTR6, GNAS, HTR2C, HTR2A |
| GO:0007611 | learning or memory | 2.79E-13 | 4.95E-10 | DRD1, PTGS2, DRD3, DRD2, DRD5, GRIN1, TH, GRIN2A, COMT, FOXP2, ATXN1, GRM4, BDNF, S100B, GRIN2B, GRIA1, GRM7, IL1B, CHRNA7, CHRNB2, CHRFAM7A, HTR2A |
| GO:0010243 | response to organic nitrogen | 4.01E-12 | 7.11E-09 | DRD1, GNAO1, DRD3, PTGS2, DRD2, DRD5, GRIN1, DRD4, NR4A2, GRIN2A, TIMP3, GNAL, PLA2G4A, SLC1A2, SLC18A2, PEMT |
| GO:0051705 | behavioral interaction between organisms | 4.94E-12 | 8.75E-09 | DRD1, GNB1L, DRD3, DRD5, DRD4, TH, GRIN1, TBX1, NPAS3, GRIN2B, PPP1R1B, IL1B, CHRNB2 |
| GO:0051050 | positive regulation of transport | 1.80E-11 | 3.18E-08 | MCHR1, STX1A, DRD1, TNF, ERBB4, ERBB3, C3, DRD2, DRD4, INHA, GDNF, IL10, AKT1, BAK1, CD38, SLC1A2, PLA2G4A, GRIN2B, APOE, GSK3B, IL1B, CHRNB2, HTR2C, IL1A, LTA, IL2 |
| GO:0014075 | response to amine stimulus | 2.14E-11 | 3.79E-08 | DRD1, DRD3, DRD2, DRD5, DRD4, GRIN1, NR4A2, GRIN2A, TIMP3, GNAL, SLC1A2, SLC18A2, PEMT |
| GO:0009628 | response to abiotic stimulus | 2.34E-11 | 4.15E-08 | MICB, DRD1, TNF, DRD3, DRD2, IL18, DRD5, KMO, TIMP3, AKT1, BAK1, SLC1A2, GRIN2B, IL1B, LTA, NPHP1, TXNIP, EGFR, NES, STX1A, NOS1, GRIN1, GRIN2A, TP53, FOXP2, SOD2, ATXN1, PLA2G4A, TIMELESS, CHRNB2, IL12B, CACNA1F, CLOCK |
| GO:0042127 | regulation of cell proliferation | 2.11E-11 | 3.75E-08 | FGF18, PTGS2, IL18, PAX6, PAWR, RBM9, IL10, BDNF, HTR1A, APOE, IL1B, CHRNA7, FGF1, CHRFAM7A, NRG1, LTA, IL1A, EGFR, TP53, INHA, CD38, DDR1, TIMELESS, IL12B, FGFR1, TNF, ERBB4, DRD3, DRD2, ERBB3, COMT, ARNT, PEMT, EGF, SCG2, IL4, TXNIP, KLF5, IL3, JARID2, CTLA4, FOXP2, SOD2, NOTCH2, PLA2G4A, NOTCH4, ADRA1A, CHRNB2, FABP7, HTR2A, IL2 |
| GO:0030534 | adult behavior | 4.27E-11 | 7.57E-08 | GABRG2, DRD1, DRD3, DRD2, GRIN1, DRD4, NR4A2, CNP, GDNF, ATXN1, SLC1A2, NPY, GRIN2D, GRM7, CHRNB2, HTR2C, CHAT |
| GO:0010033 | response to organic substance | 4.21E-11 | 7.46E-08 | TF, DRD1, TNF, ERBB4, PTGS2, DRD3, DRD2, ERBB3, SLC6A3, DRD5, TH, DRD4, COMT, XRCC1, TIMP3, IL10, AKT1, SLC1A2, GRIN2B, APOE, ATF6B, SRR, PEMT, IL1B, CHRNA7, CD4, FAS, CHRFAM7A, LTA, TXNIP, EGFR, F12, AR, GNAO1, CFB, IL1RN, GRIN1, GRIN2A, NR4A2, GNAL, DDR1, CD38, PLA2G4A, SLC18A2, CHRNB2, GNAS, GNB3, HTR2C |
| GO:0048666 | neuron development | 6.65E-11 | 1.18E-07 | FGFR1, CCKAR, DRD1, OPCML, ERBB3, DRD2, TH, PAX6, CNP, L1CAM, GDNF, BDNF, CHAT, EGFR, GNAO1, NTF3, PTPRZ1, PICK1, RTN4R, NTNG1, NR4A2, NTNG2, NUMBL, SLIT3, SOD2, S100B, MAP2, RELN, CHRNB2, CACNA1F, FEZ1 |
| GO:0001975 | response to amphetamine | 7.53E-11 | 1.34E-07 | GNAL, DRD1, DRD3, DRD2, DRD5, DRD4, GRIN1, NR4A2, GRIN2A, SLC18A2 |
| GO:0048167 | regulation of synaptic plasticity | 7.27E-11 | 1.29E-07 | DRD1, LZTS1, PTGS2, DRD2, GRIK2, DRD5, GRIN1, GRIN2A, BCAN, SYNGR1, CD38, BDNF, YWHAH, GRIN2B, APOE |
| GO:0043279 | response to alkaloid | 9.64E-11 | 1.71E-07 | DRD1, GNAO1, DRD3, DRD2, DRD5, SLC6A3, DRD4, GRIN1, GNAL, SRR, IL1B, CHRNA7, CHRNB2, CHRFAM7A, HTR2C |
| GO:0007166 | cell surface receptor linked signal transduction | 1.07E-10 | 1.90E-07 | MCHR1, FGF18, CCKAR, MPZL1, MICB, GABRB2, GRIK2, PLXNA2, GRIK3, GRIK4, GABBR1, L1CAM, AKT1, MARCO, TAAR6, HTR1A, GRIN2B, APOE, NRG1, FGF1, HTR5A, EGFR, GABRG2, IL18RAP, MAGI1, GNB1L, PICK1, HTR4, GRIN2A, INHA, PDYN, ALK, ARHGEF11, GRM5, GNAL, DDR1, GRM4, GRM3, CHRM5, GRM8, DARC, GRM7, HTR7, HTR6, GNAS, IL12B, GNB3, FGFR1, DRD1, DRD3, ERBB4, DRD2, ERBB3, C3, DRD5, DRD4, ADCYAP1, HRH1, HRH2, CNR1, CD4, EGF, TXNIP, PLP1, GNAO1, GABRA1, GABRA6, GRIN1, GPR78, FZD3, GRIA3, GRIA4, DKK4, NOTCH2, SFRP1, NPY, GSK3B, NOTCH4, MAPK8IP2, ADRA1A, HTR2C, HTR2A, IL2 |
| GO:0042417 | dopamine metabolic process | 1.34E-10 | 2.38E-07 | DRD1, DRD3, DRD2, MAOA, SLC6A3, TH, DRD4, NR4A2, GRIN2A, COMT |
| GO:0030802 | regulation of cyclic nucleotide biosynthetic process | 2.24E-10 | 3.97E-07 | MCHR1, DRD1, DRD3, DRD2, DRD5, DRD4, GABBR1, ADCYAP1, GRM4, GNAL, CHRM5, GRM3, HTR1A, GRM8, APOE, GRM7, HTR7, GNAS |
| GO:0030808 | regulation of nucleotide biosynthetic process | 2.24E-10 | 3.97E-07 | MCHR1, DRD1, DRD3, DRD2, DRD5, DRD4, GABBR1, ADCYAP1, GRM4, GNAL, CHRM5, GRM3, HTR1A, GRM8, APOE, GRM7, HTR7, GNAS |
| GO:0045761 | regulation of adenylate cyclase activity | 2.40E-10 | 4.26E-07 | MCHR1, DRD1, DRD3, DRD2, DRD5, DRD4, GABBR1, ADCYAP1, GRM4, GNAL, CHRM5, GRM3, HTR1A, GRM8, GRM7, HTR7, GNAS |
| GO:0031175 | neuron projection development | 3.57E-10 | 6.34E-07 | EGFR, CCKAR, FGFR1, GNAO1, NTF3, ERBB3, DRD2, PTPRZ1, NR4A2, NTNG1, RTN4R, PAX6, NTNG2, CNP, L1CAM, GDNF, NUMBL, SLIT3, BDNF, S100B, MAP2, RELN, CHRNB2, CACNA1F, CHAT, FEZ1 |
| GO:0030799 | regulation of cyclic nucleotide metabolic process | 3.46E-10 | 6.13E-07 | MCHR1, DRD1, DRD3, DRD2, DRD5, DRD4, GABBR1, ADCYAP1, GRM4, GNAL, CHRM5, GRM3, HTR1A, GRM8, APOE, GRM7, HTR7, GNAS |
| GO:0031279 | regulation of cyclase activity | 3.87E-10 | 6.86E-07 | MCHR1, DRD1, DRD3, DRD2, DRD5, DRD4, GABBR1, ADCYAP1, GRM4, GNAL, CHRM5, GRM3, HTR1A, GRM8, GRM7, HTR7, GNAS |
| GO:0007612 | learning | 4.00E-10 | 7.09E-07 | DRD1, DRD3, DRD2, DRD5, GRIN1, TH, GRIN2A, COMT, FOXP2, ATXN1, GRM4, GRIN2B, GRM7, CHRNB2 |
| GO:0007613 | memory | 4.29E-10 | 7.60E-07 | DRD1, PTGS2, GRIN1, TH, GRIN2A, GRIN2B, GRIA1, GRM7, IL1B, CHRNB2, CHRNA7, CHRFAM7A, HTR2A |
| GO:0019935 | cyclic-nucleotide-mediated signaling | 4.21E-10 | 7.46E-07 | MCHR1, DRD1, DRD3, DRD2, DRD5, DRD4, HTR4, GRM4, GNAL, CHRM5, HTR1A, NPY, APOE, HRH2, CNR1, HTR7, HTR6, GNAS, HTR5A |
| GO:0006140 | regulation of nucleotide metabolic process | 5.27E-10 | 9.35E-07 | MCHR1, DRD1, DRD3, DRD2, DRD5, DRD4, GABBR1, ADCYAP1, GRM4, GNAL, CHRM5, GRM3, HTR1A, GRM8, APOE, GRM7, HTR7, GNAS |
| GO:0051339 | regulation of lyase activity | 5.26E-10 | 9.32E-07 | MCHR1, DRD1, DRD3, DRD2, DRD5, DRD4, GABBR1, ADCYAP1, GRM4, GNAL, CHRM5, GRM3, HTR1A, GRM8, GRM7, HTR7, GNAS |
| GO:0030817 | regulation of cAMP biosynthetic process | 5.26E-10 | 9.32E-07 | MCHR1, DRD1, DRD3, DRD2, DRD5, DRD4, GABBR1, ADCYAP1, GRM4, GNAL, CHRM5, GRM3, HTR1A, GRM8, GRM7, HTR7, GNAS |
| GO:0001964 | startle response | 5.15E-10 | 9.12E-07 | DRD1, GRIN2B, DRD3, DRD2, SLC6A3, GRIN2D, GRIN1, GRIN2A, FABP7 |
| GO:0030814 | regulation of cAMP metabolic process | 7.10E-10 | 1.26E-06 | MCHR1, DRD1, DRD3, DRD2, DRD5, DRD4, GABBR1, ADCYAP1, GRM4, GNAL, CHRM5, GRM3, HTR1A, GRM8, GRM7, HTR7, GNAS |
| GO:0014070 | response to organic cyclic substance | 1.03E-09 | 1.83E-06 | TF, DRD1, GNAO1, DRD3, PTGS2, DRD2, DRD5, SLC6A3, GRIN1, DRD4, COMT, TIMP3, GNAL, SRR, IL1B, CHRNA7, CHRNB2, CHRFAM7A, HTR2C |
| GO:0007242 | intracellular signaling cascade | 1.49E-09 | 2.64E-06 | MCHR1, CCKAR, NRG3, RBM9, IL10, AKT1, HTR1A, APOE, PIK3C3, RAPGEF6, IL1B, CHRNA7, CHRFAM7A, FGF1, S100A1, HTR5A, LTA, EGFR, MAGI3, AR, MAGI2, PIK3C2G, GNB1L, MED12, PI4KA, HTR4, TP53, ARHGEF11, GRM5, GNAL, GRM4, CHRM5, HTR7, HTR6, GNAS, GNB3, FGFR1, DRD1, TNF, DRD3, DRD2, ERBB3, DRD5, DRD4, MYO9B, HRH1, HRH2, CNR1, EGF, SCG2, ITK, YWHAE, YWHAH, NPY, GSK3B, RGS4, NOTCH4, MAPK8IP2, ADRA1A, CHN2, PLA2G4C, HTR2C, HTR2A |
| GO:0007215 | glutamate signaling pathway | 1.49E-09 | 2.63E-06 | GRM5, GRM4, GRIN2B, GRIK2, GRIK3, GRIK4, GRIN1, GRIN2A, GRIA3, GRIA4 |
| GO:0030182 | neuron differentiation | 1.98E-09 | 3.51E-06 | FGFR1, CCKAR, DRD1, OPCML, ERBB3, DRD2, TH, PAX6, CNP, L1CAM, GDNF, BDNF, OLIG2, CHAT, EGFR, GNAO1, NTF3, PTPRZ1, PICK1, RTN4R, NTNG1, NR4A2, NEUROG1, NTNG2, NUMBL, SLIT3, SOD2, S100B, MAP2, RELN, CHRNB2, CACNA1F, FEZ1 |
| GO:0030030 | cell projection organization | 2.14E-09 | 3.80E-06 | FGFR1, CCKAR, ERBB3, DRD2, PAX6, CNP, L1CAM, GDNF, AKT1, BDNF, CHAT, KLF5, EGFR, GNAO1, NTF3, PTPRZ1, NTNG1, RPGRIP1L, NR4A2, RTN4R, NTNG2, PCM1, NUMBL, SLIT3, S100B, MAP2, RELN, CHRNB2, CACNA1F, FEZ1 |
| GO:0006873 | cellular ion homeostasis | 3.10E-09 | 5.50E-06 | TF, CCKAR, MCHR1, DRD1, DRD3, GRIK2, DRD2, GRIK3, DRD5, DRD4, HP, GCLM, BAK1, GRIN2B, APOE, SLC1A6, IL1B, CHRNA7, OLIG2, CHRFAM7A, PLP1, NTF3, GRIN1, GRIN2A, TP53, SOD2, ATXN1, CD38, CHRNB2, CACNA1F, IL2 |
| GO:0007187 | G-protein signaling, coupled to cyclic nucleotide second messenger | 3.74E-09 | 6.64E-06 | MCHR1, DRD1, DRD3, DRD2, DRD5, DRD4, HTR4, GRM4, GNAL, CHRM5, HTR1A, NPY, HRH2, CNR1, HTR7, HTR6, GNAS |
| GO:0051240 | positive regulation of multicellular organismal process | 3.71E-09 | 6.57E-06 | EGFR, F12, TF, DRD1, TNF, NOS1, ERBB4, PTGS2, GRIK2, DRD2, IL18, SLC6A3, GRIA4, ARNT, AKT1, CD38, PLA2G4A, IL1B, CHRNB2, IL12B, NRG1, IL1A, LTA, HTR2A |
| GO:0055082 | cellular chemical homeostasis | 4.45E-09 | 7.89E-06 | TF, CCKAR, MCHR1, DRD1, DRD3, GRIK2, DRD2, GRIK3, DRD5, DRD4, HP, GCLM, BAK1, GRIN2B, APOE, SLC1A6, IL1B, CHRNA7, OLIG2, CHRFAM7A, PLP1, NTF3, GRIN1, GRIN2A, TP53, SOD2, ATXN1, CD38, CHRNB2, CACNA1F, IL2 |
| GO:0042981 | regulation of apoptosis | 4.64E-09 | 8.23E-06 | RTN4, YWHAZ, TNF, PTGS2, GRIK2, ERBB3, MUTED, PAWR, TIMP3, GDNF, GCLM, IL10, AKT1, BAK1, BDNF, APOE, IL1B, PPP3CC, FAS, NRG1, LTA, IL1A, SCG2, EGFR, TXNIP, IL4, IL3, NTF3, GRIN1, TP53, NR4A2, GRIN2A, INHA, YWHAE, PROC, SOD2, ARHGEF11, NOTCH2, CD38, GRM4, PLA2G4A, SFRP1, GSK3B, IL12B, IL2, PRODH |
| GO:0043067 | regulation of programmed cell death | 6.29E-09 | 1.11E-05 | RTN4, YWHAZ, TNF, PTGS2, GRIK2, ERBB3, MUTED, PAWR, TIMP3, GDNF, GCLM, IL10, AKT1, BAK1, BDNF, APOE, IL1B, PPP3CC, FAS, NRG1, LTA, IL1A, SCG2, EGFR, TXNIP, IL4, IL3, NTF3, GRIN1, TP53, NR4A2, GRIN2A, INHA, YWHAE, PROC, SOD2, ARHGEF11, NOTCH2, CD38, GRM4, PLA2G4A, SFRP1, GSK3B, IL12B, IL2, PRODH |
| GO:0010941 | regulation of cell death | 7.01E-09 | 1.24E-05 | RTN4, YWHAZ, TNF, PTGS2, GRIK2, ERBB3, MUTED, PAWR, TIMP3, GDNF, GCLM, IL10, AKT1, BAK1, BDNF, APOE, IL1B, PPP3CC, FAS, NRG1, LTA, IL1A, SCG2, EGFR, TXNIP, IL4, IL3, NTF3, GRIN1, TP53, NR4A2, GRIN2A, INHA, YWHAE, PROC, SOD2, ARHGEF11, NOTCH2, CD38, GRM4, PLA2G4A, SFRP1, GSK3B, IL12B, IL2, PRODH |
| GO:0032990 | cell part morphogenesis | 9.30E-09 | 1.65E-05 | EGFR, CCKAR, NTF3, ERBB3, DRD2, PTPRZ1, NR4A2, NTNG1, RTN4R, RPGRIP1L, PAX6, NTNG2, CNP, L1CAM, PCM1, NUMBL, SLIT3, BAK1, BDNF, S100B, RELN, CHRNB2, CACNA1F, FEZ1 |
| GO:0007632 | visual behavior | 1.31E-08 | 2.32E-05 | ATXN1, SLC1A2, DRD1, DRD3, DRD2, DRD5, GRIN1, GRIN2A, CHRNB2, NPHP1 |
| GO:0043066 | negative regulation of apoptosis | 1.59E-08 | 2.82E-05 | YWHAZ, TNF, GRIK2, ERBB3, MUTED, GCLM, GDNF, IL10, AKT1, BDNF, APOE, IL1B, FAS, NRG1, IL1A, SCG2, IL4, EGFR, IL3, GRIN1, NR4A2, TP53, SOD2, PROC, NOTCH2, SFRP1, GSK3B, IL2 |
| GO:0051046 | regulation of secretion | 1.74E-08 | 3.09E-05 | STX1A, TNF, DRD3, ERBB3, DRD2, DRD4, INHA, GDNF, IL10, CD38, GRIN2B, GRM8, GRM7, IL1B, CHRNB2, NRG1, EGF, HTR2C, IL1A, HTR2A, IL2 |
| GO:0009719 | response to endogenous stimulus | 1.85E-08 | 3.28E-05 | DRD1, TNF, ERBB4, PTGS2, DRD3, ERBB3, DRD2, DRD5, DRD4, TH, TIMP3, IL10, AKT1, SLC1A2, PEMT, IL1B, FAS, TXNIP, AR, GNAO1, IL1RN, GRIN1, GRIN2A, NR4A2, GNAL, CD38, PLA2G4A, SLC18A2, GNAS, GNB3 |
| GO:0048858 | cell projection morphogenesis | 2.00E-08 | 3.54E-05 | EGFR, CCKAR, NTF3, ERBB3, DRD2, PTPRZ1, NR4A2, NTNG1, RTN4R, RPGRIP1L, PAX6, NTNG2, CNP, L1CAM, PCM1, NUMBL, SLIT3, BDNF, S100B, RELN, CHRNB2, CACNA1F, FEZ1 |
| GO:0032225 | regulation of synaptic transmission, dopaminergic | 1.99E-08 | 3.54E-05 | DRD1, PTGS2, DRD3, DRD2, DRD4, CHRNB2, GDNF |
| GO:0043069 | negative regulation of programmed cell death | 2.13E-08 | 3.77E-05 | YWHAZ, TNF, GRIK2, ERBB3, MUTED, GCLM, GDNF, IL10, AKT1, BDNF, APOE, IL1B, FAS, NRG1, IL1A, SCG2, IL4, EGFR, IL3, GRIN1, NR4A2, TP53, SOD2, PROC, NOTCH2, SFRP1, GSK3B, IL2 |
| GO:0060548 | negative regulation of cell death | 2.27E-08 | 4.02E-05 | YWHAZ, TNF, GRIK2, ERBB3, MUTED, GCLM, GDNF, IL10, AKT1, BDNF, APOE, IL1B, FAS, NRG1, IL1A, SCG2, IL4, EGFR, IL3, GRIN1, NR4A2, TP53, SOD2, PROC, NOTCH2, SFRP1, GSK3B, IL2 |
| GO:0031280 | negative regulation of cyclase activity | 2.24E-08 | 3.98E-05 | MCHR1, GRM4, GNAL, CHRM5, GRM3, HTR1A, DRD3, GRM8, DRD2, GRM7, DRD4, GABBR1 |
| GO:0007194 | negative regulation of adenylate cyclase activity | 2.24E-08 | 3.98E-05 | MCHR1, GRM4, GNAL, CHRM5, GRM3, HTR1A, DRD3, GRM8, DRD2, GRM7, DRD4, GABBR1 |
| GO:0051350 | negative regulation of lyase activity | 2.24E-08 | 3.98E-05 | MCHR1, GRM4, GNAL, CHRM5, GRM3, HTR1A, DRD3, GRM8, DRD2, GRM7, DRD4, GABBR1 |
| GO:0050801 | ion homeostasis | 2.32E-08 | 4.12E-05 | TF, CCKAR, MCHR1, DRD1, DRD3, GRIK2, DRD2, GRIK3, DRD5, DRD4, HP, GCLM, BAK1, GRIN2B, APOE, SLC1A6, IL1B, CHRNA7, OLIG2, CHRFAM7A, PLP1, NTF3, GRIN1, GRIN2A, TP53, SOD2, ATXN1, CD38, CHRNB2, CACNA1F, IL2 |
| GO:0006575 | cellular amino acid derivative metabolic process | 2.23E-08 | 3.96E-05 | DRD1, DRD3, DRD2, MAOA, SLC6A3, TH, DRD4, NR4A2, GRIN2A, GSTT1, KMO, COMT, GCLM, SOD2, GSS, GSR, PLA2G4A, PEMT, TPH1 |
| GO:0048878 | chemical homeostasis | 2.43E-08 | 4.30E-05 | TF, MCHR1, CCKAR, DRD1, ERBB4, DRD3, GRIK2, DRD2, GRIK3, DRD5, DRD4, HP, GCLM, BAK1, MALL, GRIN2B, APOE, SLC1A6, IL1B, CHRNA7, OLIG2, CHRFAM7A, LPL, PLP1, NTF3, GRIN1, GRIN2A, TP53, SOD2, ATXN1, CD38, PLA2G4A, CHRNB2, CACNA1F, IL2 |
| GO:0051952 | regulation of amine transport | 2.40E-08 | 4.26E-05 | DRD1, TNF, DRD3, DRD2, DRD4, CHRNB2, HTR2C, GDNF, LTA, HTR2A |
| GO:0019725 | cellular homeostasis | 3.24E-08 | 5.74E-05 | TF, MCHR1, CCKAR, DRD1, DRD3, GRIK2, DRD2, GRIK3, DRD5, DRD4, MUTED, HP, GCLM, BAK1, GSR, GRIN2B, APOE, SLC1A6, IL1B, CHRNA7, OLIG2, CHRFAM7A, PLP1, NTF3, GRIN1, GRIN2A, TP53, SOD2, ATXN1, CD38, CHRNB2, CACNA1F, IL2 |
| GO:0006584 | catecholamine metabolic process | 3.21E-08 | 5.69E-05 | DRD1, DRD3, DRD2, MAOA, SLC6A3, TH, DRD4, NR4A2, GRIN2A, COMT |
| GO:0034311 | diol metabolic process | 3.21E-08 | 5.69E-05 | DRD1, DRD3, DRD2, MAOA, SLC6A3, TH, DRD4, NR4A2, GRIN2A, COMT |
| GO:0009712 | catechol metabolic process | 3.21E-08 | 5.69E-05 | DRD1, DRD3, DRD2, MAOA, SLC6A3, TH, DRD4, NR4A2, GRIN2A, COMT |
| GO:0030900 | forebrain development | 3.49E-08 | 6.18E-05 | CCKAR, DRD1, GNAO1, ERBB4, DRD2, SLC6A3, GRIN1, SLC6A4, RPGRIP1L, PAX6, BCAN, YWHAE, FOXP2, NUMBL, SLC1A2, RELN, CHRNB2, FABP7 |
| GO:0051051 | negative regulation of transport | 3.90E-08 | 6.91E-05 | TNF, GNAO1, NOS1, DRD3, PTGS2, ERBB3, DRD2, DRD4, INHA, IL10, AKT1, GRM7, IL1B, EGF, NRG1, LTA, HTR2A |
| GO:0051960 | regulation of nervous system development | 4.03E-08 | 7.14E-05 | RTN4, MAG, PHOX2B, TF, LZTS1, NTF3, DRD3, DRD2, GRIN1, TP53, PAX6, RTN4R, TTL, NUMBL, BDNF, YWHAH, ATN1, APOE, CHRNB2, EGF |
| GO:0048812 | neuron projection morphogenesis | 4.26E-08 | 7.55E-05 | EGFR, CCKAR, NTF3, ERBB3, DRD2, PTPRZ1, NR4A2, NTNG1, PAX6, RTN4R, NTNG2, CNP, L1CAM, NUMBL, SLIT3, BDNF, S100B, RELN, CHRNB2, CACNA1F, FEZ1 |
| GO:0007623 | circadian rhythm | 4.25E-08 | 7.54E-05 | EGFR, DRD1, EGR3, TIMELESS, DRD3, ERBB3, DRD2, HTR7, CHRNB2, PER3, CLOCK |
| GO:0007409 | axonogenesis | 4.38E-08 | 7.77E-05 | CCKAR, NTF3, ERBB3, DRD2, PTPRZ1, NR4A2, NTNG1, PAX6, RTN4R, NTNG2, CNP, L1CAM, NUMBL, SLIT3, BDNF, S100B, RELN, CHRNB2, CACNA1F, FEZ1 |
| GO:0018958 | phenol metabolic process | 4.24E-08 | 7.51E-05 | DRD1, DRD3, DRD2, MAOA, SLC6A3, TH, DRD4, NR4A2, GRIN2A, COMT |
| GO:0048168 | regulation of neuronal synaptic plasticity | 5.54E-08 | 9.83E-05 | DRD1, GRIN2B, GRIK2, DRD2, APOE, DRD5, GRIN1, GRIN2A, BCAN, SYNGR1 |
| GO:0007212 | dopamine receptor signaling pathway | 5.66E-08 | 1.00E-04 | GNAL, DRD1, GNAO1, DRD3, DRD2, DRD5, DRD4, GNAS |
| GO:0000904 | cell morphogenesis involved in differentiation | 8.79E-08 | 1.56E-04 | CCKAR, NTF3, ERBB3, DRD2, PTPRZ1, NR4A2, NTNG1, RTN4R, PAX6, NTNG2, CNP, L1CAM, NUMBL, SLIT3, BDNF, S100B, NOTCH4, RELN, CHRNB2, CACNA1F, FN1, FEZ1 |
| GO:0035176 | social behavior | 8.79E-08 | 1.56E-04 | NPAS3, GNB1L, DRD3, DRD4, GRIN1, IL1B, TBX1, CHRNB2 |
| GO:0051966 | regulation of synaptic transmission, glutamatergic | 8.79E-08 | 1.56E-04 | EGFR, DRD1, PTGS2, GRIK2, DRD2, GRIK3, GRIA4, HTR2A |
| GO:0031328 | positive regulation of cellular biosynthetic process | 1.07E-07 | 1.89E-04 | DRD1, TNF, DRD3, DRD5, PAX6, TBP, PAWR, GDNF, IL10, ARNT, AKT1, HRH1, APOE, IL1B, CD4, IL1A, IL4, KLF5, EGFR, IL3, KLF6, SOX10, AR, NTF3, GRIN1, MED12, TP53, NR4A2, TBX1, ARHGEF11, SOD2, ATXN1, MED7, PLA2G4A, YWHAH, NOTCH4, IL12B, CLOCK, IL2 |
| GO:0008284 | positive regulation of cell proliferation | 1.10E-07 | 1.95E-04 | FGFR1, FGF18, TNF, ERBB4, DRD3, PTGS2, DRD2, IL18, PAX6, ARNT, HTR1A, IL1B, CHRNA7, CHRFAM7A, EGF, NRG1, FGF1, SCG2, IL4, KLF5, EGFR, IL3, FOXP2, CD38, PLA2G4A, NOTCH4, CHRNB2, IL12B, IL2, HTR2A |
| GO:0031646 | positive regulation of neurological system process | 1.18E-07 | 2.09E-04 | EGFR, TF, DRD1, TNF, PTGS2, ERBB4, GRIK2, CHRNB2, GRIA4, LTA |
| GO:0043085 | positive regulation of catalytic activity | 1.17E-07 | 2.08E-04 | CCKAR, DRD1, TNF, DRD3, DRD2, DRD5, DRD4, GCLM, GDNF, ADCYAP1, AKT1, BAK1, HRH1, APOE, IL1B, CHRNA7, CD4, CHRFAM7A, NRG1, EGF, EGFR, GNAO1, PICK1, NR4A2, TP53, GRM5, GNAL, GRM4, HTR7, RELN, GNAS, HTR2C, HTR2A, IL2 |
| GO:0048511 | rhythmic process | 1.21E-07 | 2.15E-04 | EGFR, EGR3, DRD1, DRD3, ERBB3, DRD2, GRIN2A, INHA, PLA2G4A, TIMELESS, GRIN2B, HTR7, CHRNB2, PER3, CHAT, CLOCK |
| GO:0050767 | regulation of neurogenesis | 1.28E-07 | 2.28E-04 | RTN4, MAG, PHOX2B, LZTS1, DRD3, NTF3, DRD2, GRIN1, TP53, PAX6, RTN4R, TTL, NUMBL, BDNF, YWHAH, ATN1, APOE, CHRNB2 |
| GO:0042592 | homeostatic process | 1.41E-07 | 2.50E-04 | TF, MCHR1, CCKAR, DRD1, ERBB4, DRD3, DRD2, GRIK2, GRIK3, DRD5, DRD4, MUTED, HP, GCLM, AKT1, GSR, BAK1, MALL, GRIN2B, APOE, SLC1A6, IL1B, CHRNA7, FAS, OLIG2, CHRFAM7A, IL1A, LPL, PLP1, NTF3, GRIN1, TP53, GRIN2A, INHA, SOD2, ATXN1, CD38, PLA2G4A, CHRNB2, CACNA1F, IL2, HTR2A |
| GO:0007626 | locomotory behavior | 1.47E-07 | 2.60E-04 | IL4, DRD1, GNAO1, DRD3, DRD2, SLC6A3, GRIN1, DRD4, TH, NR4A2, CNP, GDNF, IL10, SOD2, ATXN1, NPAS3, GRIN2D, SLC18A2, IL1B, RELN, CHRNB2, CHAT, SCG2 |
| GO:0048667 | cell morphogenesis involved in neuron differentiation | 1.56E-07 | 2.77E-04 | CCKAR, NTF3, ERBB3, DRD2, PTPRZ1, NR4A2, NTNG1, PAX6, RTN4R, NTNG2, CNP, L1CAM, NUMBL, SLIT3, BDNF, S100B, RELN, CHRNB2, CACNA1F, FEZ1 |
| GO:0009891 | positive regulation of biosynthetic process | 1.55E-07 | 2.75E-04 | DRD1, TNF, DRD3, DRD5, PAX6, TBP, PAWR, GDNF, IL10, ARNT, AKT1, HRH1, APOE, IL1B, CD4, IL1A, IL4, KLF5, EGFR, IL3, KLF6, SOX10, AR, NTF3, GRIN1, MED12, TP53, NR4A2, TBX1, ARHGEF11, SOD2, ATXN1, MED7, PLA2G4A, YWHAH, NOTCH4, IL12B, CLOCK, IL2 |
| GO:0006576 | biogenic amine metabolic process | 1.75E-07 | 3.11E-04 | DRD1, DRD3, DRD2, MAOA, SLC6A3, DRD4, TH, GRIN2A, NR4A2, KMO, COMT, PLA2G4A, PEMT, TPH1 |
| GO:0009611 | response to wounding | 1.79E-07 | 3.17E-04 | TF, YWHAZ, TNF, C3, ERBB3, DRD5, CRP, TIMP3, DTNBP1, IL10, AKT1, SLC1A2, HRH1, BLOC1S3, IL1B, NRG1, IL1A, SCG2, FN1, F12, KLF6, IL18RAP, C4A, CFB, C4B, IL1RN, GRIN2A, SOD2, PROC, APOL3, NOTCH2, DARC, PLA2G4C |
| GO:0044093 | positive regulation of molecular function | 1.87E-07 | 3.31E-04 | CCKAR, DRD1, TNF, DRD3, DRD2, DRD5, DRD4, GCLM, GDNF, IL10, ADCYAP1, AKT1, BAK1, HRH1, APOE, IL1B, CHRNA7, CD4, CHRFAM7A, NRG1, EGF, IL4, EGFR, GNAO1, PICK1, NR4A2, TP53, GRM5, GNAL, GRM4, HTR7, RELN, GNAS, HTR2C, HTR2A, IL2 |
| GO:0050805 | negative regulation of synaptic transmission | 1.95E-07 | 3.46E-04 | CD38, DRD1, PTGS2, GRIK2, DRD2, GRIK3, DRD5, HTR2A |
| GO:0048169 | regulation of long-term neuronal synaptic plasticity | 1.95E-07 | 3.46E-04 | DRD1, GRIN2B, GRIK2, DRD2, DRD5, GRIN1, GRIN2A, SYNGR1 |
| GO:0042391 | regulation of membrane potential | 2.23E-07 | 3.96E-04 | DRD1, PLP1, NTF3, GRIK2, GRIK3, DRD4, GRIN1, GRIN2A, GCLM, SOD2, ATXN1, BAK1, GRIN2B, SLC1A6, CHRNB2, OLIG2 |
| GO:0007270 | nerve-nerve synaptic transmission | 2.37E-07 | 4.21E-04 | DRD1, DRD3, GRM8, DRD2, GABRA6, DRD5, GRIN2D, TH, GRIN1 |
| GO:0010647 | positive regulation of cell communication | 2.37E-07 | 4.19E-04 | FGF18, DRD1, TNF, DRD3, PTGS2, ERBB4, ERBB3, GRIK2, L1CAM, ARNT, IL1B, CD4, EGF, LTA, EGFR, IL4, IL3, GRIA4, APOL3, GRM4, NOTCH2, MAPK8IP2, CHRNB2, RELN, IL2 |
| GO:0042053 | regulation of dopamine metabolic process | 2.53E-07 | 4.49E-04 | DRD1, SLC6A3, DRD4, NR4A2, CHRNB2, COMT |
| GO:0051048 | negative regulation of secretion | 2.57E-07 | 4.55E-04 | TNF, DRD3, ERBB3, DRD2, GRM7, DRD4, IL1B, INHA, NRG1, EGF, IL10 |
| GO:0048585 | negative regulation of response to stimulus | 2.52E-07 | 4.47E-04 | DRD1, MICB, DRD3, DRD2, SLC6A3, GRIN1, CTLA4, IL10, AKT1, APOE, IL1B, CHRNA7, CHRFAM7A, FABP7, IL2 |
| GO:0007631 | feeding behavior | 2.61E-07 | 4.63E-04 | MCHR1, CCKAR, DRD1, BDNF, NPY, GRIN2B, DRD2, GRM7, TH, GRIN1, CHRNB2, HTR2C |
| GO:0000902 | cell morphogenesis | 2.71E-07 | 4.80E-04 | CCKAR, ERBB3, DRD2, PAX6, CNP, L1CAM, BDNF, FN1, EGFR, NTF3, PTPRZ1, NTNG1, RPGRIP1L, NR4A2, RTN4R, NTNG2, PCM1, ARHGEF11, SLIT3, NUMBL, S100B, NOTCH4, RELN, CHRNB2, CACNA1F, FEZ1 |
| GO:0009416 | response to light stimulus | 3.29E-07 | 5.83E-04 | EGFR, DRD1, DRD3, DRD2, DRD5, GRIN1, TP53, GRIN2A, ATXN1, AKT1, SLC1A2, CHRNB2, IL12B, CACNA1F, CLOCK, NPHP1 |
| GO:0009314 | response to radiation | 3.87E-07 | 6.87E-04 | EGFR, NES, DRD1, DRD3, DRD2, DRD5, GRIN1, TP53, GRIN2A, SOD2, ATXN1, AKT1, BAK1, SLC1A2, CHRNB2, IL12B, CACNA1F, CLOCK, NPHP1 |
| GO:0032101 | regulation of response to external stimulus | 3.85E-07 | 6.83E-04 | F12, DRD1, DRD3, PTGS2, DRD2, C3, SLC6A3, GRIN1, IL10, PROC, PLA2G4A, NPY, APOE, CHRNA7, CHRFAM7A, FABP7, IL2, SCG2 |
| GO:0043271 | negative regulation of ion transport | 3.97E-07 | 7.03E-04 | NOS1, TNF, GNAO1, PTGS2, DRD2, DRD4, LTA, HTR2A |
| GO:0050806 | positive regulation of synaptic transmission | 5.16E-07 | 9.14E-04 | EGFR, DRD1, TNF, PTGS2, ERBB4, GRIK2, CHRNB2, GRIA4, LTA |
| GO:0060341 | regulation of cellular localization | 5.13E-07 | 9.10E-04 | STX1A, TNF, DRD3, DRD2, DRD4, INHA, GDNF, IL10, UHMK1, CD38, GRIN2B, GRM8, GSK3B, GRM7, IL1B, CHRNB2, EGF, HTR2C, IL1A, HTR2A, IL2 |
| GO:0042069 | regulation of catecholamine metabolic process | 5.59E-07 | 9.91E-04 | DRD1, SLC6A3, DRD4, NR4A2, CHRNB2, COMT |
| GO:0043523 | regulation of neuron apoptosis | 5.55E-07 | 9.85E-04 | GRM4, BDNF, TNF, NTF3, ERBB3, GRIK2, GRIN1, NR4A2, TP53, GCLM, GDNF, LTA, SOD2 |
| GO:0032989 | cellular component morphogenesis | 5.99E-07 | 0.001061 | CCKAR, ERBB3, DRD2, PAX6, CNP, L1CAM, BAK1, BDNF, FN1, EGFR, NTF3, PTPRZ1, NTNG1, RPGRIP1L, NR4A2, RTN4R, NTNG2, PCM1, ARHGEF11, SLIT3, NUMBL, S100B, NOTCH4, RELN, CHRNB2, CACNA1F, FEZ1 |
| GO:0051970 | negative regulation of transmission of nerve impulse | 7.49E-07 | 0.001329 | CD38, DRD1, PTGS2, GRIK2, DRD2, GRIK3, DRD5, HTR2A |
| GO:0051588 | regulation of neurotransmitter transport | 1.01E-06 | 0.001784 | STX1A, DRD1, DRD3, GRM8, DRD2, DRD4, HTR2C, GDNF |
| GO:0019933 | cAMP-mediated signaling | 1.01E-06 | 0.001783 | GRM4, GNAL, MCHR1, CHRM5, DRD1, HTR1A, DRD3, DRD2, HTR7, DRD5, DRD4, GNAS, HTR5A |
| GO:0051971 | positive regulation of transmission of nerve impulse | 1.04E-06 | 0.001837 | EGFR, DRD1, TNF, PTGS2, ERBB4, GRIK2, CHRNB2, GRIA4, LTA |
| GO:0015844 | monoamine transport | 1.03E-06 | 0.001832 | CHRM5, DRD1, SLC6A3, PICK1, SLC6A4, SLC18A2, SLC18A1 |
| GO:0008306 | associative learning | 1.03E-06 | 0.001832 | DRD1, GRIN2B, DRD2, GRM7, DRD5, GRIN1, CHRNB2 |
| GO:0060134 | prepulse inhibition | 1.10E-06 | 0.001947 | DRD1, DRD3, DRD2, SLC6A3, GRIN1, FABP7 |
| GO:0051584 | regulation of dopamine uptake | 1.09E-06 | 0.001931 | DRD1, DRD3, DRD2, DRD4, GDNF |
| GO:0051940 | regulation of catecholamine uptake during transmission of nerve impulse | 1.09E-06 | 0.001931 | DRD1, DRD3, DRD2, DRD4, GDNF |
| GO:0045471 | response to ethanol | 1.12E-06 | 0.00198 | GRIN2B, DRD3, DRD2, APOE, SLC6A3, TH, DRD4, GRIN1, PEMT, GRIN2A, CHRNB2 |
| GO:0032102 | negative regulation of response to external stimulus | 1.13E-06 | 0.002 | DRD1, DRD3, DRD2, APOE, SLC6A3, GRIN1, CHRNA7, CHRFAM7A, FABP7, IL10, IL2 |
| GO:0021537 | telencephalon development | 1.30E-06 | 0.002296 | SLC1A2, DRD1, ERBB4, DRD2, GRIN1, PAX6, RPGRIP1L, BCAN, RELN, YWHAE, FOXP2 |
| GO:0008542 | visual learning | 1.33E-06 | 0.002364 | ATXN1, DRD1, DRD3, DRD2, DRD5, GRIN1, GRIN2A, CHRNB2 |
| GO:0043269 | regulation of ion transport | 1.57E-06 | 0.002791 | MCHR1, DRD1, GNAO1, TNF, NOS1, PTGS2, DRD3, DRD2, DRD4, AKT1, BAK1, LTA, HTR2A |
| GO:0033555 | multicellular organismal response to stress | 1.59E-06 | 0.002818 | DRD1, BDNF, NOS1, GRIN2B, GRIK2, GRM7, DRD4, RELN, COMT |
| GO:0051173 | positive regulation of nitrogen compound metabolic process | 1.63E-06 | 0.002891 | DRD1, TNF, DRD3, DRD5, PAX6, TBP, COMT, GDNF, IL10, ARNT, AKT1, HRH1, APOE, IL1B, IL4, KLF5, EGFR, IL3, KLF6, SOX10, AR, NTF3, GRIN1, MED12, TP53, NR4A2, TBX1, SOD2, ARHGEF11, ATXN1, MED7, YWHAH, NOTCH4, CLOCK, IL2 |
| GO:0001505 | regulation of neurotransmitter levels | 1.73E-06 | 0.003061 | GRM4, STX1A, NOS1, MAOA, SYN3, TH, SYN2, SLC6A4, COMT, GAD1, CHAT |
| GO:0031645 | negative regulation of neurological system process | 1.75E-06 | 0.003094 | CD38, DRD1, PTGS2, GRIK2, DRD2, GRIK3, DRD5, HTR2A |
| GO:0014073 | response to tropane | 2.18E-06 | 0.00387 | DRD1, DRD3, DRD2, SLC6A3, DRD5, DRD4, CHRNB2 |
| GO:0042220 | response to cocaine | 2.18E-06 | 0.00387 | DRD1, DRD3, DRD2, SLC6A3, DRD5, DRD4, CHRNB2 |
| GO:0008344 | adult locomotory behavior | 2.22E-06 | 0.003934 | ATXN1, DRD1, DRD2, GRIN2D, DRD4, GRIN1, NR4A2, CNP, GDNF, CHAT |
| GO:0007188 | G-protein signaling, coupled to cAMP nucleotide second messenger | 2.24E-06 | 0.003971 | MCHR1, GRM4, GNAL, CHRM5, DRD1, HTR1A, DRD3, DRD2, HTR7, DRD5, DRD4, GNAS |
| GO:0060284 | regulation of cell development | 2.55E-06 | 0.004529 | RTN4, MAG, PHOX2B, LZTS1, DRD3, NTF3, DRD2, GRIN1, TP53, PAX6, RTN4R, TTL, NUMBL, BDNF, YWHAH, ATN1, APOE, CHRNB2 |
| GO:0006916 | anti-apoptosis | 2.73E-06 | 0.004837 | IL3, YWHAZ, TNF, MUTED, GDNF, IL10, SOD2, AKT1, NOTCH2, BDNF, SFRP1, APOE, GSK3B, IL1B, FAS, NRG1, IL1A, IL2 |
| GO:0048015 | phosphoinositide-mediated signaling | 3.17E-06 | 0.005621 | GRM5, CCKAR, DRD1, HRH1, PIK3C2G, NPY, DRD2, DRD5, PIK3C3, PI4KA, HTR2C, HTR2A |
| GO:0051580 | regulation of neurotransmitter uptake | 3.21E-06 | 0.005693 | DRD1, DRD3, DRD2, DRD4, GDNF |
| GO:0007191 | activation of adenylate cyclase activity by dopamine receptor signaling pathway | 3.21E-06 | 0.005693 | GNAL, DRD1, DRD3, DRD5, GNAS |
| GO:0008285 | negative regulation of cell proliferation | 4.37E-06 | 0.007746 | TNF, PTGS2, DRD2, JARID2, CTLA4, TP53, PAX6, COMT, PAWR, IL10, SOD2, DDR1, NOTCH2, BDNF, APOE, PEMT, ADRA1A, IL1B, IL12B, FABP7, IL1A, LTA, IL2, SCG2 |
| GO:0055066 | di-, tri-valent inorganic cation homeostasis | 5.08E-06 | 0.009007 | CCKAR, MCHR1, TF, DRD1, DRD3, DRD2, GRIK2, DRD5, GRIN1, DRD4, HP, SOD2, BAK1, CD38, APOE, IL1B, CHRNA7, CACNA1F, CHRFAM7A, IL2 |
| GO:0033238 | regulation of cellular amine metabolic process | 5.31E-06 | 0.009422 | DRD1, SLC6A3, DRD4, NR4A2, CHRNB2, COMT |
| GO:0009991 | response to extracellular stimulus | 6.66E-06 | 0.011811 | CCKAR, NES, MICB, PTGS2, CFB, NR4A2, TP53, ENSA, TIMP3, SOD2, AKT1, CD38, PLA2G4A, SLC1A2, NPY, PEMT, IL1B, LTA |
| GO:0045664 | regulation of neuron differentiation | 6.66E-06 | 0.011804 | RTN4, MAG, PHOX2B, LZTS1, GRIN1, PAX6, RTN4R, TTL, NUMBL, BDNF, YWHAH, ATN1, APOE, CHRNB2 |
| GO:0007169 | transmembrane receptor protein tyrosine kinase signaling pathway | 8.41E-06 | 0.014901 | EGFR, TXNIP, FGF18, FGFR1, MPZL1, ERBB4, ERBB3, GRIK2, PICK1, ALK, ADCYAP1, AKT1, DDR1, MAPK8IP2, CD4, FGF1, EGF, NRG1 |
| GO:0030005 | cellular di-, tri-valent inorganic cation homeostasis | 1.00E-05 | 0.017791 | CCKAR, MCHR1, TF, DRD1, DRD3, DRD2, GRIK2, DRD5, GRIN1, DRD4, HP, BAK1, CD38, APOE, IL1B, CHRNA7, CACNA1F, CHRFAM7A, IL2 |
| GO:0007186 | G-protein coupled receptor protein signaling pathway | 1.03E-05 | 0.018186 | MCHR1, CCKAR, DRD1, DRD3, GABRB2, DRD2, C3, GRIK3, DRD5, DRD4, GABBR1, ADCYAP1, AKT1, TAAR6, HRH1, HTR1A, HRH2, APOE, CNR1, HTR5A, GABRG2, GNAO1, GABRA1, GNB1L, GABRA6, PICK1, HTR4, GPR78, FZD3, PDYN, ARHGEF11, GRM5, GNAL, GRM4, GRM3, CHRM5, NPY, DARC, GRM8, HTR7, GRM7, HTR6, ADRA1A, GNAS, GNB3, HTR2C, HTR2A, IL2 |
| GO:0006874 | cellular calcium ion homeostasis | 1.15E-05 | 0.020383 | CCKAR, MCHR1, DRD1, DRD3, DRD2, GRIK2, DRD5, DRD4, GRIN1, BAK1, CD38, APOE, IL1B, CHRNA7, CACNA1F, CHRFAM7A, IL2 |
| GO:0030003 | cellular cation homeostasis | 1.18E-05 | 0.020888 | CCKAR, MCHR1, TF, DRD1, DRD3, DRD2, GRIK2, DRD5, GRIN1, DRD4, TP53, HP, BAK1, CD38, APOE, IL1B, CHRNA7, CACNA1F, CHRFAM7A, IL2 |
| GO:0070201 | regulation of establishment of protein localization | 1.30E-05 | 0.02306 | AKT1, TNF, DRD3, DRD2, GSK3B, DRD4, IL1B, EGF, UHMK1, IL10, LTA, IL1A, IL2 |
| GO:0051241 | negative regulation of multicellular organismal process | 1.42E-05 | 0.025208 | F12, DRD1, TNF, PTGS2, DRD2, GRIK2, DRD5, GRIK3, IL10, PROC, CD38, APOE, CHRNA7, CHRFAM7A, IL2, HTR2A |
| GO:0055074 | calcium ion homeostasis | 1.59E-05 | 0.028148 | CCKAR, MCHR1, DRD1, DRD3, DRD2, GRIK2, DRD5, DRD4, GRIN1, BAK1, CD38, APOE, IL1B, CHRNA7, CACNA1F, CHRFAM7A, IL2 |
| GO:0055080 | cation homeostasis | 1.68E-05 | 0.029846 | CCKAR, MCHR1, TF, DRD1, DRD3, DRD2, GRIK2, DRD5, GRIN1, DRD4, TP53, HP, SOD2, BAK1, CD38, APOE, IL1B, CHRNA7, CACNA1F, CHRFAM7A, IL2 |
| GO:0014072 | response to isoquinoline alkaloid | 1.71E-05 | 0.030298 | GNAO1, DRD3, DRD2, GRIN1, SRR, IL1B |
| GO:0043278 | response to morphine | 1.71E-05 | 0.030298 | GNAO1, DRD3, DRD2, GRIN1, SRR, IL1B |
| GO:0010975 | regulation of neuron projection development | 2.00E-05 | 0.035448 | RTN4, MAG, LZTS1, YWHAH, APOE, GRIN1, RTN4R, CHRNB2, TTL, NUMBL |
| GO:0045428 | regulation of nitric oxide biosynthetic process | 2.06E-05 | 0.036444 | EGFR, AKT1, HRH1, TNF, IL1B, IL10, SOD2 |
| GO:0051249 | regulation of lymphocyte activation | 2.13E-05 | 0.037684 | IL4, IL18, CTLA4, INHA, PAWR, IL10, CD38, BLOC1S3, IL1B, CHRNB2, CD4, FAS, IL12B, IL2 |
| GO:0007193 | inhibition of adenylate cyclase activity by G-protein signaling | 2.20E-05 | 0.038923 | MCHR1, GRM4, GNAL, CHRM5, HTR1A, DRD3, DRD2, DRD4 |
| GO:0051047 | positive regulation of secretion | 2.53E-05 | 0.044928 | CD38, STX1A, TNF, GRIN2B, DRD2, CHRNB2, INHA, HTR2C, GDNF, IL10, IL1A, IL2 |
| GO:0006875 | cellular metal ion homeostasis | 2.60E-05 | 0.046078 | CCKAR, MCHR1, DRD1, DRD3, DRD2, GRIK2, DRD5, DRD4, GRIN1, BAK1, CD38, APOE, IL1B, CHRNA7, CACNA1F, CHRFAM7A, IL2 |
| GO:0031667 | response to nutrient levels | 2.76E-05 | 0.048871 | CCKAR, MICB, NES, PTGS2, CFB, TP53, ENSA, TIMP3, SOD2, AKT1, CD38, PLA2G4A, NPY, PEMT, IL1B, LTA |
| GO:0043068 | positive regulation of programmed cell death | 2.75E-05 | 0.048816 | TNF, PTGS2, GRIK2, PAWR, TIMP3, IL10, AKT1, BAK1, APOE, IL1B, PPP3CC, FAS, LTA, TXNIP, GRIN1, GRIN2A, TP53, INHA, YWHAE, ARHGEF11, NOTCH2, CD38, PLA2G4A, IL12B, PRODH |

Supplemental Table S6. The connection of novel candidate genes with schizophrenia and/or nicotine addiction

| **Gene Symbol** | **Gene Name** | **Available studies supporting the connection of the gene with schizophrenia and/or nicotine addiction**^a^ | |
| --- | --- | --- | --- |
|  |  | **Schizophrenia** | **Nicotine addiction** |
| FYN | FYN proto-oncogene, Src family tyrosine kinase | 12670706, 17417065, 19501919, 17460065, 19468241, 18552348, 27784625 | 25516494, 27784625 |
| PRKCA | protein kinase C, alpha | 19786960, 21281445 | 11813828, 20407865 |
| SUMO1 | small ubiquitin-like modifier 1 | 22126837 | 15582157 |
| APP | amyloid beta precursor protein | 25889058 | 14622092 |
| CAMK2A | calcium/calmodulin-dependent protein kinase II alpha | 21084551, 19289156, 25297099, 23942359 | --^b^ |
| CTNNB1 | catenin beta 1 | 26027441 | 25680692 |
| GRB2 | growth factor receptor bound protein 2 | 21195589, 17202467 | -- |
| CALM1 | Calmodulin 1 (phosphorylase kinase, delta) | 24927284^c^ | 17205118^c^ |
| CSNK2A1 | casein kinase 2, alpha 1 polypeptide | - | -- |
| ELAVL1 | ELAV like RNA binding protein 1 | -- | 27575817^c^ |
| VHL | von Hippel-Lindau tumor suppressor | -- | 22842216, 16892044 |

^a^ Literatures are designated by PMIDs, i.e., the identifiers for publication in PubMed. A gene followed by one or more publications indicates the gene has been found to be related to schizophrenia and/or nicotine addiction in these studies.

^b^ No supporting study available.

^c^ The diseases reported in these publications are not schizophrenia disease or nicotine addiction, but other complex diseases like autism (24927284), drug addiction (17205118) and pulmonary sarcoidosis (27575817).
